# Epidemic threshold in pairwise models for clustered networks: closures and fast correlations

**DOI:** 10.1007/s00285-019-01380-1

**Published:** 2019-05-11

**Authors:** Rosanna C. Barnard, Luc Berthouze, Péter L. Simon, István Z. Kiss

**Affiliations:** 1 Department of Mathematics, School of Mathematical and Physical Sciences, University of Sussex, Falmer, Brighton, BN1 9QH UK; 2 Centre for Computational Neuroscience and Robotics, University of Sussex, Falmer, Brighton, BN1 9QH UK; 3 Institute of Mathematics, Eötvös Loránd University Budapest, Budapest, Hungary; 4 Numerical Analysis and Large Networks Research Group, Hungarian Academy of Sciences, Budapest, Hungary

**Keywords:** Network, Epidemic, Pairwise model, Clustering, Correlation, Fast variables, 34E10, 92D30, 34D20, 90B10

## Abstract

The epidemic threshold is probably the most studied quantity in the modelling of epidemics on networks. For a large class of networks and dynamics, it is well studied and understood. However, it is less so for clustered networks where theoretical results are mostly limited to idealised networks. In this paper we focus on a class of models known as pairwise models where, to our knowledge, no analytical result for the epidemic threshold exists. We show that by exploiting the presence of fast variables and using some standard techniques from perturbation theory we are able to obtain the epidemic threshold analytically. We validate this new threshold by comparing it to the threshold based on the numerical solution of the full system. The agreement is found to be excellent over a wide range of values of the clustering coefficient, transmission rate and average degree of the network. Interestingly, we find that the analytical form of the threshold depends on the choice of closure, highlighting the importance of model selection when dealing with real-world epidemics. Nevertheless, we expect that our method will extend to other systems in which fast variables are present.

## Introduction

Epidemic dynamics on networks, being susceptible-infected-susceptible (SIS), susceptible-infected-recovered (SIR) or otherwise, are often modelled as continuous time Markov chains with discrete but extremely large state spaces of order $m^{N}$, where *m* denotes the number of different disease statuses (e.g. $m=2$ for SIS and $m=3$ for SIR) and *N* stands for the number of nodes in the network. This makes the analysis of the resulting exact system almost impossible, except for some specific network topologies such as the fully connected network, networks with considerable structural symmetry or networks with few nodes (Kiss et al. 2017; Holme 2017).

Often, this problem is dealt with by focusing on mean-field models where the goal is to derive, often heuristically, a system of ordinary or integro-differential equations that describe (non-Markovian) epidemics for some average quantities, such as the expected number of nodes in various states, the expected number of links in various states or the expected number of star-like structures (focusing on a node and all of its neighbours). These methods usually rely on closures to break the dependency on higher-order moments (e.g. the expected number of nodes in a state depends on the expected number of links in certain states and so on). Such an approach has led to a number of models including heterogeneous or degree-based mean-field (Pastor-Satorras and Vespignani 2001; Pastor-Satorras et al. 2015), pairwise (Rand 1999; Keeling 1999), effective-degree (Lindquist et al. 2011), edge-based compartmental (Miller et al. 2012) and message passing (Karrer and Newman 2010a), to name a few. These models essentially differ in the choice of variables over which the averaging is done. Perhaps the most compact model with the fewest number of equations is the edge-based compartmental model (Miller and Volz 2013) which is valid for heterogeneous networks with Markovian SIR epidemics, although extensions of this model for arbitrary infection and recovery processes are possible (Sherborne et al. 2018).

Pairwise models have been extremely popular and the very first model for regular networks and SIR epidemics (Rand 1999; Keeling 1999) has been generalised to heterogeneous networks (Eames and Keeling 2002), preferentially mixing networks (Eames and Keeling 2002), directed (Sharkey et al. 2006) and weighted networks (Rattana et al. 2013), adaptive networks (Gross et al. 2006; Kiss et al. 2012; Szabó-Solticzky et al. 2016), and structured networks (House et al. 2009) among others. Perhaps this is due to the relative simplicity and transparency of the pairwise model, whereby variables have a straightforward interpretation and a basic understanding of the network and epidemic dynamics coupled with good bookkeeping leads to valid and analytically tractable model equations. Pairwise models have been successfully used to derive analytically the epidemic threshold and final epidemic size, with these results mostly limited to networks without clustering. The propensity of contacts to cluster, i.e. two neighbours of a node being neighbours of one another, is known to lead to many complications, and modelling epidemics on clustered networks using analytically tractable mean-field models is still limited to networks with specific structural features (House et al. 2009; Newman 2009; Miller 2009a, b; Karrer and Newman 2010b; Volz et al. 2011; Ritchie et al. 2016). However, using approaches borrowed from percolation theory (Miller 2009b) and focusing more on the stochastic process itself (Trapman 2007a), some results have been obtained. For example, Miller (2009b) showed that for the SIR epidemic on clustered networks with heterogeneous degree distributions, the basic reproduction number is given by

1.1 $$\begin{aligned} R_0=\frac{\langle k^2-k\rangle }{\langle k\rangle }T-\frac{2\langle n_{\triangle }\rangle }{\langle k\rangle }T^2+\cdots , \end{aligned}$$

where $\langle k^i \rangle$ stands for the *i*th moment of the degree distribution, *T* is the probability of infection spreading across a link connecting an infected to a susceptible node and $\langle n_{\triangle }\rangle$ denotes the average number of triangles that a node belongs to. The first positive term in Eq. (1.1) corresponds to the threshold for configuration-type networks without clustering. The second term in Eq. (1.1), which is negative, shows that clustering reduces the epidemic threshold when compared to the unclustered case, the contribution of the remaining terms being of a smaller order.

For pairwise models, clustering first manifests itself by requiring a different and more complex closure, which makes the analysis of the resulting system, even for regular networks and SIR dynamics, challenging. Furthermore, it turns out that such a closure may in fact fail to conserve pair-level relations and may not accurately reflect the early growth of quantities such as closed loops of three with all nodes being infected (House and Keeling 2010). Such considerations have led to an improved closure being developed in an effort to keep as many true features of the exact epidemic process as possible (House and Keeling 2010). In this paper we focus on the classic pairwise model for regular networks with clustering, using both the simplest closure and a variant of the improved closure. We show that by working with two fast variables, corresponding to correlations between neigbouring nodes during the epidemic, we can analytically determine the epidemic threshold as an asymptotic expansion in terms of the global clustering coefficient $\phi$, defined in Sect. 2.1.

The use of fast variables is not new (Keeling 1999; Juher et al. 2013; Llensa et al. 2014; Britton et al. 2016; Eames 2008). However, in many cases the epidemic threshold has only been obtained numerically and it was framed in terms of a growth-rate-based threshold, which is equivalent to the basic reproduction number at the critical point. Eames (2008) considered a hybrid pairwise model incorporating random and clustered contacts, with the analysis focusing on the growth-rate-based threshold. Eames (2008) derived a number of results, some analytic (the critical clustering coefficient for which an epidemic can take off) and some semi-analytic, and showed, in agreement with most studies, that clustering inhibits the spread of the epidemic when compared to an equivalent network without clustering but with equivalent parameter values governing the epidemic process. However, no analytic expression for the epidemic threshold was provided.

More recently, Li et al. (2018) calculated the epidemic threshold in a pairwise model for clustered networks with closures based on the number of links in a motif, rather than nodes. This led to

1.2 $$\begin{aligned} R_0=\frac{(n-1)\tau }{\tau +\gamma +\tau \phi }, \end{aligned}$$

where *n* is the average number of links per node, $\phi$ is the global clustering coefficient, and $\tau$ and $\gamma$ are the infection and recovery rates, respectively. The expression above can be expanded in terms of the clustering coefficient $\phi$ to give

1.3 $$\begin{aligned} R_0=\frac{(n-1)\tau }{\tau +\gamma }\left( \frac{1}{1+ \phi \frac{\tau }{\tau +\gamma }}\right) \simeq \frac{(n-1)\tau }{\tau +\gamma }\left( 1-\phi \frac{\tau }{\tau +\gamma }+\cdots \right) , \end{aligned}$$

which again demonstrates that clustering reduces the epidemic threshold.

Building on these results, and effectively extending the work by Keeling (1999) and Eames (2008), our paper presents a method to determine the epidemic threshold analytically and applies it in the context of pairwise models with two different closures for clustered networks. The paper is structured as follows. In Sect. 2 we outline the model with closures for unclustered and clustered networks discussed in Sect. 3. In Sect. 4 we briefly review existing results and approaches for the pairwise model with the simple closure and then focus on the correlation structure in terms of fast variables, showing that the epidemic threshold can be expressed via the solution of a cubic polynomial. This key solution is determined numerically and analytically as an asymptotic expansion in terms of the clustering coefficient. In Sect. 5 we show that our approach extends to a compact version of the improved closure, thus validating and generalising our approach. Finally, we conclude with a discussion of the results, including comparing the threshold to other known results and touching upon a number of possible extensions.

## Model formulation

### The network

We begin by considering a population of *N* individuals with its contact structure described by an undirected network with adjacency matrix $G=(g_{ij})_{i,j=1,2,\dots , N}$ where $g_{ij}=1$ if nodes *i* and *j* are connected and zero otherwise. Self-loops are excluded, so $g_{ii}=0$ and $g_{ij}=g_{ji}$ for all $i,j=1,2, \dots N$. The network is static and regular, such that each individual has exactly *n* edges or links. The sum over all elements of *G* is defined as $||G||=\sum _{i,j}g_{ij}$. Hence, the number of doubly counted links in the network is $||G||=nN$. More importantly, using simple matrix operations on *G*, we can calculate the global clustering coefficient of the network

2.1 $$\begin{aligned} \phi =\frac{trace(G^{3})}{||G^{2}||-trace(G^{2})}, \end{aligned}$$

where $trace(G^{3})$ yields six times the number of closed triples or loops of length three (uniquely counted) and $||G^{2}||-trace(G^{2})$ is twice the number of triples (open and closed, also uniquely counted).

### SIR dynamics

The standard SIR epidemic dynamics on a network is considered. The dynamics are driven by two processes: (a) infection and (b) recovery from infection. Infection can spread from an infected and infectious node to any of its susceptible neighbours and this is modelled as a Poisson point process with per-link infection rate $\tau$. Infectious nodes recover from infection at constant rate $\gamma$.

### The unclosed pairwise model

Let $A_{i}$ equal 1 if the individual at node *i* is of type *A* and equal zero otherwise. Then single nodes (singles) of type *A* can be counted as $[A]=\sum _{i}A_{i}$, pairs of nodes (pairs) of type $A-B$ can be counted as $[AB]=\sum _{i,j}A_{i}B_{j}g_{ij}$ and triples of nodes (triples) of type $A-B-C$ can be counted as $[ABC]=\sum _{i,j,k}A_{i}B_{j}C_{k}g_{ij}g_{jk}$. This method of counting means that pairs are counted once in each direction, so $[AB]=[BA]$, and [*AA*] is even. Using this notation to keep track of singles, pairs and triples leads to the following system of pairwise equations describing the SIR epidemic on networks:

2.2 $$\begin{aligned} \dot{[S]}= & {} -\tau [SI], \end{aligned}$$

2.3 $$\begin{aligned} \dot{[I]}= & {} \tau [SI] -\gamma [I], \end{aligned}$$

2.4 $$\begin{aligned} \dot{[SI]}= & {} \tau ([SSI]-[ISI]-[SI])-\gamma [SI], \end{aligned}$$

2.5 $$\begin{aligned} \dot{[SS]}= & {} -2\tau [SSI], \end{aligned}$$

2.6 $$\begin{aligned} \dot{[II]}= & {} 2\tau ([ISI]+[SI])-2\gamma [II]. \end{aligned}$$

**Fig. 1:**
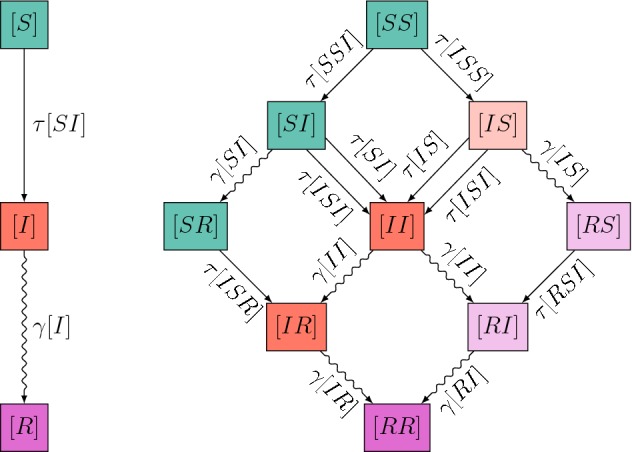
Flow diagrams showing the flux between compartments of singles (left) and compartments of pairs (right) for the SIR pairwise model. In the compartments of pairs, straight arrows denote infections coming from within the pair (with a rate depending on a pair) or from outside the pair (with a rate depending on a triple), and curved arrows denote a recovery. The colour indicates the status of the “first” node in the pair. Symmetry allows us to conclude that some of the variables (see lighter shaded variables on the right hand side of the pairs diagram) must equal their symmetric version (e.g. $[RS]=[SR]$), so we do not need to directly calculate both quantities

We note that Eqs. (2.4)–(2.6) contain triples but evolution equations for these are not given. To determine solutions of the system, we must find a way to account for these triples in terms of pairs and singles, a method referred to as *closing the system*. The system above is exact before a closure is applied. This means that it can be derived directly from the exact stochastic epidemic model on the network, given by a continuous time Markov Chain, without making any approximations [a precise proof for the SIS epidemic was given by Taylor et al. (2012)]. The flow between compartments and the rates of the SIR pairwise model are illustrated in Fig. 1. The system given above only contains dynamically relevant variables, i.e. those that emerge naturally but following a strict bookkeeping rule, and those that appear when a chosen closure for the triples is considered.

## Closures

**Fig. 2:**
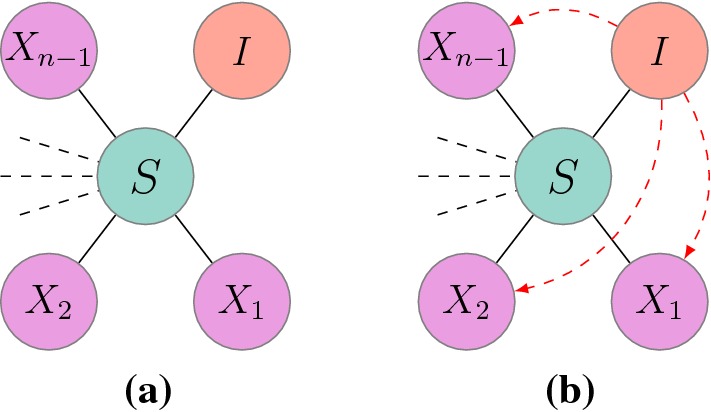
General setup for a central susceptible node with a given infected neighbour for **a** unclustered and **b** clustered regular networks with degree *n*. Dashed arrows indicate that the infected node may be connected to the other neighbours of the central susceptible node. Random variables $X_1, X_2, \dots , X_{n-1}$ take values from the set $\{S, I, R\}$

A quick inspection of the unclosed pairwise system (2.2)–(2.6) reveals that only triples of type [*ASI*] need closing, with $A\in \{S, I\}$. These triples, as well as triples of type [*RSI*], are illustrated in Fig. 2 for unclustered and clustered networks.

### Closure for unclustered networks

First, we consider the situation depicted in Fig. 2a. We aim to find an approximation for the distribution of the random variables $X_i$ which take values from the set $\{S, I, R\}$. Several observations can be made. The expected number of $A-S$ type links is [*AS*] and the total number of links emanating from susceptible nodes counted across the whole network is *n*[*S*]. Hence, the most straightforward approximation would be to assume that $X_{i}$, with $i=1,2,\dots ,n-1$, are independent and identically Bernoulli distributed random variables with probability $p_{A|S-I}^{uc}=\frac{[AS]}{n[S]}$, where $p_{A|S-I}^{uc}$ stands for the probability that a neighbour of a susceptible node already connected to an infected node will be in state *A*, provided that the network is unclustered. Averaging across the whole network leads to the closure

3.1 $$\begin{aligned}{}[ASI]=[SI](n-1)p_{A|S-I}^{uc}=\frac{n-1}{n}\frac{[AS][SI]}{[S]}. \end{aligned}$$

It is important to note that the new closed system, obtained upon using Eq. (3.1) in system (2.2)–(2.6), is effectively an approximation of the exact pairwise model (2.2)–(2.6) and one should question if closure (3.1) conserves the properties of the stochastic process and those of the counting on the network. For example, it is expected that in the closed system the number of nodes is conserved, i.e. $[S]+[I]+[R]=N$. Furthermore, the number of pairs of different types must sum to *nN*. More subtle conditions refer to the conservation of link types at node level ($[SS]+[SI]+[SR]=n[S]$) and pair level ($[SSI]+[ISI]+[RSI]=(n-1)[SI]$), respectively. It turns out that the closure for unclustered networks (3.1) conserves these relations (Kiss et al. 2017). Finally, the validity of closures can be empirically assessed by looking at the initial growth rate of the number of open and closed triples, where the number of open triples comprised of three infectious nodes should grow differently to the number of such closed triples. Of course such subtle tests are usually preceded by direct comparisons between the numerical solution of the closed pairwise system and explicit stochastic network simulations for a range of parameters. Such tests initially focus on prevalence of infection and final epidemic size but may include expected number of pairs.

### Closures for clustered networks

#### Simple closure

The presence of closed loops of length three, as illustrated in Fig. 2b, introduces some complications. Namely, a neighbour of the central susceptible node that is itself connected to an infected neighbour of the central node is less likely to be susceptible due to the added pressure from the infected neighbour, when compared to the case when the force of infection is distributed evenly, as it is the case for the closure for unclustered networks (3.1). More precisely, the epidemic process on the network displays clear correlations. Cator and Van Mieghem (2014) have shown that the exact SIS and SIR epidemics on networks are non-negatively correlated in the sense that ${\mathbf {P}}(I_{i}I_{j})\ge {\mathbf {P}}(I_i){\mathbf {P}}(I_j)$. Here, ${\mathbf {P}}(I_iI_j)$ represents the probability that nodes *i* and *j*, connected by a link, are both infected, while ${\mathbf {P}}(I_i)$ stands for the probability of node *i* being infected. For this result to hold, all processes must be Markovian and infection rates across all links and recovery rates of all nodes have to be fixed a priori. Using the pairwise model for an *SIS* epidemic on an unclustered network with closure (3.1), it has been shown that the same correlation is preserved when averaging at the population level (Kiss et al. 2017). While the proof has not been extended to the pairwise SIR model, intuitively we expect to find the same correlation structure. Based on these observations, we assume that the correlation structure in exact SIS and SIR epidemics on networks averaged at the population level is maintained. Hence, the inequalities

3.2 $$\begin{aligned}{}[SI]\le n[S]\frac{[I]}{N}, \,\,\, [II]\ge n[I]\frac{[I]}{N}, \,\,\, \text {and} \,\,\, [SS]\ge n[S]\frac{[S]}{N}, \end{aligned}$$

hold, where [*AB*] and [*A*] with $A,B\in \{S,I\}$ represent the expected counts of pairs and singles of the corresponding types taken from the exact model, i.e., the continuous time full Markov chain.

Intuitively, this means that as the epidemic spreads on the network, infected nodes are more likely to have neighbours which are themselves infected (either those that infected the node or were infected by it), and at the ‘front’ of the epidemic we would expect to observe a ‘sea’ of susceptible nodes alongside a ‘front’ of links between susceptible and infected nodes that drives the epidemic. Hence, clustering and correlations need to be accounted for and a new $p_{A|S-I}^{c}$ for clustered networks needs to be defined. This has been done by Keeling (1999) [see also work by Rand (1999) and Keeling et al. (1997)] and relies on a correlation factor, $C_{AB}$, that is able to capture the propensity that two nodes connected by a link are in states *A* and *B*, respectively. This is given by

3.3 $$\begin{aligned} C_{AB}=\frac{[AB]}{n[A]\frac{[B]}{N}}, \end{aligned}$$

where $A,B\in \{S,I\}$. This effectively compares the expected number of edges of type [*AB*] to what its value would be if nodes were labelled at random with [*A*] nodes of type *A* and [*B*] nodes of type *B*. If $C_{AB}>1$, then nodes of type *A* and *B* are positively correlated, whereas if nodes of type *A* and *B* are negatively correlated, $C_{AB}<1$. As expected, $C_{AB}=1$ means that nodes are effectively labelled as type *A* or *B* at random. Equation (3.2) implies that

3.4 $$\begin{aligned} C_{SI}\le 1, \,\,\, C_{II}\ge 1 \,\,\, \text {and} \,\,\, C_{SS}\ge 1. \end{aligned}$$

We can modify $p_{A|S-I}^{uc}=\frac{[AS]}{n[S]}$ to reflect these observations, leading to $p_{A|S-I}^{c}=\frac{[AS]}{n[S]}C_{AI}$. However, before the closure can be written, open and closed loops need to be treated separately. In order to do this, we split the closure based on whether the neighbour whose state is to be determined is part of a closed loop of three nodes and thus in direct contact with an infectious node, or not. This leads to

3.5 $$\begin{aligned} p_{A|S-I}^{c}= {\left\{ \begin{array}{ll} p_{A|S-I}^{uc} &{} \text {with probability}\ (1-\phi ),\\ p_{A|S-I}^{uc}C_{AI}&{} \text {with probability}\ \phi , \end{array}\right. } \end{aligned}$$

where $\phi$ is defined in Eq. (2.1). With this in mind, the closure can be derived by averaging equation (3.1) over the unclustered and clustered parts of the network. This leads to

3.6 $$\begin{aligned}{}[ASI]= & {} (1-\phi )(n-1)[SI]p_{A|S-I}^{uc}+\phi (n-1)[SI]p_{A|S-I}^{uc}C_{AI} \end{aligned}$$

3.7 $$\begin{aligned}= & {} \frac{(n-1)}{n}\frac{[AS][SI]}{[S]}\left( (1-\phi )+\phi \frac{N[AI]}{n[A][I]}\right) . \end{aligned}$$

This same closure has been derived by Keeling et al. (1997) and Keeling (1999). Framing $p_{A|S-I}^{uc}$ and $p_{A|S-I}^{c}$ more generally and independently of the network type, i.e. simply considering $p_A$, the following statement holds:

##### Proposition 1

Consider a closure of the following form $[ASI]=(n-1)[SI]p_{A}$. If $\sum _{A}p_{A}=1$, where *A* is taken over all possible states, then $\sum _{A}[ASI]=(n-1)[SI]$.

##### Proof

$$\sum _{A}[ASI]=(n-1)[SI]\sum _{A}p_{A}=(n-1)[SI]$$. $$\square $$

#### Improved closure

We note that while $p_{A|S-I}^{uc}$ satisfies the above proposition, $p_{A|S-I}^{c}$ does not. In particular, we find

$$\begin{aligned} \sum _{A}[ASI]&=\sum _{A}(n-1)[SI]p_{A|S-I}^{uc}=\sum _{A}(n-1)[SI]\frac{[AS]}{n[S]}\\&=\frac{(n-1)[SI]}{n[S]}\sum _{A}[AS]=\frac{(n-1)[SI]}{n[S]}n[S]=(n-1)[SI]. \end{aligned}$$

However, for the clustered part of the network this is not the case. We find that

$$\begin{aligned} \sum _{A}[ASI]&=\sum _{A}(n-1)[SI]p_{A|S-I}^{c}=\sum _{A}(n-1)[SI] \frac{[AS]}{n[S]}\frac{N[AI]}{n[A][I]}\\&=\frac{(n-1)N[SI]}{n^2[S][I]}\sum _{A}\frac{[AS][AI]}{[A]}, \end{aligned}$$

which does not result in the desired $(n-1)[SI]$. This can be corrected in a straightforward way by defining

3.8 $$\begin{aligned} p_{A|S-I}^{c_{new}}= {\left\{ \begin{array}{ll} p_{A|S-I}^{uc} &{} \text {with probability}\ (1-\phi ),\\ \frac{p_{A|S-I}^{c}}{\sum _{a}p_{a|S-I}^{c}} &{} \text {with probability}\ \phi . \end{array}\right. } \end{aligned}$$

Hence we can now write

$$\begin{aligned} \sum _{A}[ASI]&=\sum _{A}\left( (1-\phi )[ASI]+\phi [ASI]\right) \\&=(1-\phi )(n-1)[SI]\sum _{A}p_{A|S-I}^{uc}+\phi (n-1)[SI] \sum _{A}p_{A|S-I}^{c_{new}}\\&=(1-\phi )(n-1)[SI]\sum _{A}\frac{[AS]}{n[S]}+\phi (n-1)[SI]\sum _{A} \frac{p_{A|S-I}^{c}}{\sum _{a}p_{a|S-I}^{c}}\\&=(1-\phi )(n-1)[SI]\frac{1}{n[S]}\sum _{A}[AS]+\phi (n-1)[SI]\\&=(1-\phi )(n-1)[SI]+\phi (n-1)[SI]\\ {}&=(n-1)[SI], \end{aligned}$$

as required. It is informative to investigate the relationship between the various probability models that lead to different closures. This is summarised in the following proposition.

##### Proposition 2

For closures applied across the clustered part of the network and assuming that the number of nodes in state *R* is negligible, it follows that

3.9 $$\begin{aligned} p_{S|S-I}^{c_{new}}=\frac{[SS][I]}{[SS][I]+[II][S]} ,\,\,\, p_{S|S-I}^{c}=\frac{[SS]}{n[S]}\frac{N[SI]}{n[S][I]},\,\,\, p_{S|S-I}^{uc}=\frac{[SS]}{n[S]}, \end{aligned}$$

and

3.10 $$\begin{aligned} p_{S|S-I}^{c} \le p_{S|S-I}^{uc} \,\,\, \text {and} \,\,\, p_{S|S-I}^{c_{new}} \le p_{S|S-I}^{uc}. \end{aligned}$$

##### Proof

All three probabilities follow from their definitions and assuming that $A\in \{S,I\}$. Since $S-I$ links are negatively correlated (3.2), it follows that $C_{SI}=\frac{N[SI]}{n[S][I]}\le 1$ and as a result

3.11 $$\begin{aligned} p_{S|S-I}^{c}=\frac{[SS]}{n[S]}C_{SI}\le \frac{[SS]}{n[S]} =p_{S|S-I}^{uc}. \end{aligned}$$

While $p_{S|S-I}^{c}$ has a natural interpretation (it is a simple discounted variant of the probability from the unclustered network case and takes into account the observation that if the neighbour of a central susceptible node is connected to one of the infected neighbours of the same node then it is less likely that the node in question is susceptible), the interpretation of $p_{S|S-I}^{c_{new}}$ is less obvious. A close inspection reveals that $p_{S|S-I}^{c_{new}}$ can be rewritten as

3.12 $$\begin{aligned} p_{S|S-I}^{c_{new}}=\frac{[SS][I]}{[SS][I]+[II][S]}= \frac{[SS]}{[SS]+[II]\frac{[S]}{[I]}}. \end{aligned}$$

However, combining $[SI] \le n[S]\frac{[I]}{N}$ with $[I] \le \frac{N}{n}\frac{[II]}{[I]}$, as given in Eq. (3.2), leads to $[SI] \le [II]\frac{[S]}{[I]}$. Finally, using the relation $[SI] \le [II]\frac{[S]}{[I]}$ in Eq. (3.12) yields

3.13 $$\begin{aligned} p_{S|S-I}^{c_{new}}=\frac{[SS]}{[SS]+[II]\frac{[S]}{[I]}} \le \frac{[SS]}{[SS]+[SI]}=\frac{[SS]}{n[S]}=p_{S|S-I}^{uc}. \end{aligned}$$

Equation (3.13) illustrates that as expected $p_{S|S-I}^{c_{new}} \le p_{S|S-I}^{uc}$. Again, this simply shows that for clustered networks and for the setup in Fig. 2, it is less likely to find neighbours who are susceptible compared with the unclustered network case. $\square$

Taking into account the new way of defining $p_{A|S-I}^{c_{new}}$, the improved closure yields

3.14 $$\begin{aligned}{}[ASI]&=(1-\phi )[ASI]+\phi [ASI] \nonumber \\&= (1-\phi )(n-1)[SI]\frac{[AS]}{n[S]}+\phi (n-1)[SI]\frac{\frac{[AS]}{n[S]}C_{AI}}{\sum _{a}p_{a|S-I}^{c}}\nonumber \\&= (1-\phi )\frac{(n-1)}{n}\frac{[AS][SI]}{[S]}+\phi (n-1)[SI] \frac{\frac{[AS]}{n[S]}\frac{N[AI]}{n[A][I]}}{\sum _{a} \frac{[aS]}{n[S]}\frac{N[aI]}{n[a][I]}}\nonumber \\&=(1-\phi )\frac{(n-1)}{n}\frac{[AS][SI]}{[S]}+\phi (n-1) \frac{[AS][SI][IA]}{[A]\sum _{a}\frac{[aS][aI]}{[a]}}\nonumber \\&=(n-1)\left( (1-\phi )\frac{[AS][SI]}{n[S]}+\phi \frac{[AS][SI][IA]}{[A]\sum _{a}[aS][aI]/[a]}\right) . \end{aligned}$$

We finally note that the closures rely heavily on the assumption of how the states of the neighbours are distributed, and the assumption of independent and identically Bernoulli-distributed variables is a strong one. For clustered networks in particular, we have illustrated different ways of incorporating correlations induced by closed cycles of length three. Despite these seemingly strong assumptions, it is known that the pairwise model for unclustered networks is equivalent to the edge-based compartmental equivalent on configuration networks (Miller and Kiss 2014; Kiss et al. 2017) and the latter has been shown to be the limiting system of the stochastic network epidemic model (Decreusefond et al. 2012; Janson et al. 2014). For clustered networks we are not aware of such results.

## Results for the pairwise model with the simple closure

### Background

Using the simple closure for clustered networks (3.7), and writing $\xi =\frac{(n-1)}{n}$, we obtain the following closed pairwise model equations describing an SIR epidemic on a clustered regular network of *N* individuals with degree *n*:

4.1 $$\begin{aligned} \dot{[S]}&=-\tau [SI], \end{aligned}$$

4.2 $$\begin{aligned} \dot{[I]}&=\tau [SI]-\gamma [I], \end{aligned}$$

4.3 $$\begin{aligned} \dot{[SI]}&=-(\tau +\gamma )[SI]+\tau \xi \frac{[SS][SI]}{[S]} \left( (1-\phi )+\phi \frac{N[SI]}{n[S][I]}\right) \nonumber \\&\quad - \tau \xi \frac{[SI]^{2}}{[S]}\left( (1-\phi )+ \phi \frac{N[II]}{n[I]^{2}}\right) , \end{aligned}$$

4.4 $$\begin{aligned} \dot{[SS]}&=-2\tau \xi \frac{[SS][SI]}{[S]}\left( (1-\phi )+\phi \frac{N[SI]}{n[S][I]}\right) , \end{aligned}$$

4.5 $$\begin{aligned} \dot{[II]}&=2\tau [SI]-2\gamma [II]+2\tau \xi \frac{[SI]^{2}}{[S]} \left( (1-\phi )+\phi \frac{N[II]}{n[I]^{2}}\right) . \end{aligned}$$

For model equations (4.1)–(4.5), the basic reproductive ratio ($R_{0}$) is considered by Keeling (1999). Starting from the evolution equation of the expected number of infectious nodes leads to

$$\begin{aligned} \dot{[I]}&=\tau [SI]-\gamma [I] \\&=\left( \frac{\beta [S]}{N}C_{SI}-\gamma \right) [I], \end{aligned}$$

where $C_{SI}$ is defined in Eq. (3.3). Taking into account that $\tau n=\beta$ and that initially $[S]\simeq N$, Keeling (1999) claimed that $R_{0}=C_{SI}\beta /\gamma$. It is important to note that this $R_0$ is not the classical $R_0$ in the sense of describing the expected number of new infections produced by a typical infectious individual when introduced into a fully susceptible population. Rather it can be thought of as a growth-rate-based threshold, and has the same properties as the classical $R_0$ when both are exactly one. In what follows, we will simply refer to it as *R* (Eames 2008; Kiss et al. 2012).

In order to determine *R* explicitly, Keeling (1999) considered the early behaviour of $C_{SI}$ and found that this variable is given by the ordinary differential equation (ODE)

4.6 $$\begin{aligned} \dot{C_{SI}}=-\tau \left( C_{SI}+C_{SI}^{2}-n\xi (C_{SI}-C_{SI}^{2})(1-\phi )+n\xi C_{SI}^{2}\phi \frac{[I]C_{II}}{N}\right) . \end{aligned}$$

However, the ODE above depends on the behaviour of $[I]C_{II}/N$ and Keeling (1999) showed that

4.7 $$\begin{aligned} \frac{[I]C_{II}}{N}\longrightarrow \frac{2\tau C_{SI}}{\gamma +\beta C_{SI}-2\xi \beta C_{SI}^{2}\phi }. \end{aligned}$$

Considering the quasi-equilibrium of $C_{SI}$, referred to as $C_{SI}^{*}$, in Eq. (4.6) together with the expression for $[I]C_{II}/N$ in Eq. (4.7), one finds that $C_{SI}^{*}$ is given by

4.8 $$\begin{aligned} 1+C_{SI}^{*}-n\xi (1-C_{SI}^{*})(1-\phi )+\frac{2\beta \xi \phi {C_{SI}^{*}}^{2}}{\gamma +\beta C_{SI}^{*}-2\xi \beta {C_{SI}^{*}}^{2}\phi }=0. \end{aligned}$$

Hence, *R* can be calculated as $C_{SI}^{*}\beta /\gamma$, at least numerically. Variables such as $C_{SI}$ and $C_{II}$ describe the correlations between the states of neighbouring nodes on the network as the epidemic unfolds and these have been studied numerically by Keeling (1999).

For model equations (4.1)–(4.5) and when there is no clustering in the network ($\phi =0$), a further simplification of Eq. (4.8) can be achieved (Keeling 1999). To determine $R=C_{SI}^{*}\beta /\gamma$ in this case, one can simply solve

4.9 $$\begin{aligned} 1+C_{SI}^{*}-n\xi (1-C_{SI}^{*})=0 \end{aligned}$$

to find $C_{SI}^{*}=\frac{n-2}{n}$ and thus $R=\frac{(n-2)\tau }{\gamma }$.

Unfortunately when $\phi \ne 0$, according to our knowledge, the quasi-equilibrium values can only be determined numerically via Eq. (4.8). In what follows, we show that by working with two new variables, $\alpha =[SI]/[I]$ and $\delta =[II]/[I]$, which are still closely linked to the correlations formed during the spreading process, it is possible to obtain the epidemic threshold as the solution of a cubic equation and, more importantly, we show that this solution can be approximated by an asymptotic expansion in powers of $\phi$.

### Epidemic threshold

Consider the initial phase of an infection invading an entirely susceptible population in the pairwise model, described by Eqs. (4.1)–(4.5). We find that

4.10 $$\begin{aligned} \dot{[I]}=\tau [SI]-\gamma [I]=\gamma [I]\left( \frac{\tau [SI]}{\gamma [I]}-1\right) . \end{aligned}$$

We know the quantity $\gamma [I]$ remains non-negative regardless of time in the epidemic process, and we choose to consider the epidemic threshold in terms of $\frac{[SI]}{[I]}$. This leads to $R=\frac{\tau [SI]}{\gamma [I]}$. When $R>1$ an epidemic will occur, and when $R<1$ the epidemic will die out. Although we know the values of $\tau$ and $\gamma$, to determine if an epidemic will occur *a priori*, we require further knowledge about the quantity $\frac{[SI]}{[I]}$ at some initial time close to $t=0$. At $t=0$ or at the disease-free steady state, both [*SI*] and [*I*] are zero and hence their ratio is ill-defined. Furthermore, gaining knowledge about $\frac{[SI]}{[I]}$ will involve $\frac{[II]}{[I]}$ and this term suffers from the same problem, being ill-defined at $t=0$. While this is similar to the approach taken by Keeling (1999), we focus on the variables $\frac{[SI]}{[I]}$ and $\frac{[II]}{[I]}$, and we motivate our choice below. The problem of finding the epidemic threshold can be dealt with in at least two other different but equivalent ways. First, one can carry out a simple linear stability analysis of the disease-free steady state as is shown in Appendices B and C. Second, the threshold can also be computed as the largest eigenvalue of the next generation matrix, see Sect. 6. However, in both cases, the variables [*SI*] / [*I*] and [*II*] / [*I*] turn out to play a key role and their values for small times need to be determined.

### Fast variables with the simple closure

**Fig. 3:**
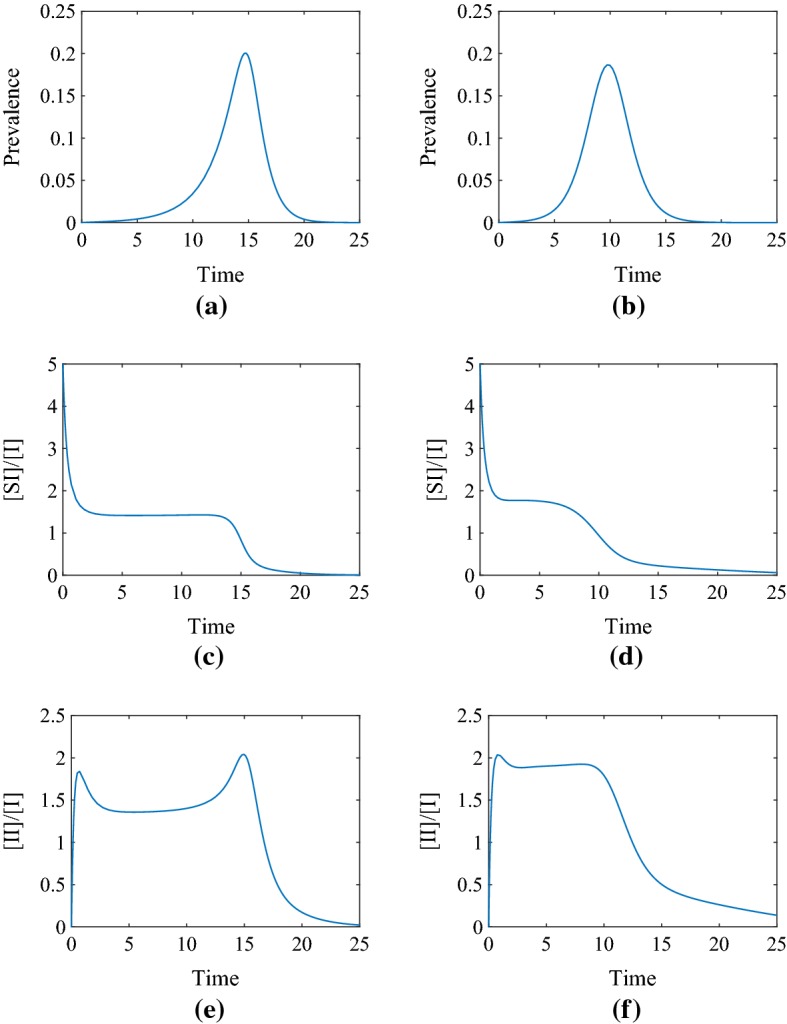
Illustration of the dynamics of prevalence, [*I*] / *N*, over time (**a**, **b**), compared to that of $\alpha =\frac{[SI]}{[I]}$ (**c**, **d**) and $\delta =\frac{[II]}{[I]}$ (**e**, **f**) for the pairwise model with the simple (left column) and the improved (right column) closures. Parameter values are $N=10{,}000$, $n=5$, $\phi =0.5$ and $\tau =\gamma =1$

To circumvent the problem of the ill-defined variables above, we exploit the fact that $\alpha :=\frac{[SI]}{[I]}$ and $\delta :=\frac{[II]}{[I]}$ are fast variables when compared to the time course of the epidemic. Figure 3 shows clearly that $\alpha$ and $\delta$ are fast compared to the epidemic process and that they quickly converge to a quasi-equilibrium. Hence, at early times, $\alpha$ and $\delta$ attain their quasi-equilibrium values, and these are the values that can be used to compute the epidemic threshold.

We continue by deriving differential equations for the variables $\alpha =\frac{[SI]}{[I]}$ and $\delta =\frac{[II]}{[I]}$. Differentiating $\alpha$ and $\delta$ and using Eqs. (4.1)–(4.5) leads to

4.11 $$\begin{aligned} \frac{d\alpha }{dt}= & {} -\tau \alpha +\tau \xi n(1-\phi )\alpha +\tau \xi \phi \alpha ^{2}-\tau \xi \frac{1}{n}\phi \alpha ^{2}\delta -\tau \alpha ^{2}, \end{aligned}$$

4.12 $$\begin{aligned} \frac{d\delta }{dt}= & {} 2\tau \alpha -\gamma \delta +2\tau \xi \frac{1}{n} \phi \alpha ^{2}\delta -\tau \alpha \delta . \end{aligned}$$

We note that this approach has already been exploited by Juher et al. (2013), Llensa et al. (2014) and Britton et al. (2016), with the authors focusing on combinations of SIS, SIR and SEIR models without demography and rewiring of $S-I$ links to $S-S$ links. In all these papers, systems of fast variables are derived and analysed in detail to gain information about the epidemic threshold and the stability of the disease-free or endemic steady states.

#### Global stability of the steady state

The analysis of system (4.11)–(4.12) is carried out in detail by Trapman (2007b) (see Appendix A of this paper). The only caveat there is that the global stability of the unique steady state was not confirmed, leaving the possibility of the existence of a limit cycle. Below, we sketch the main ideas of the proof and provide some extra results by using the Bendixson criterion.

**Fig. 4:**
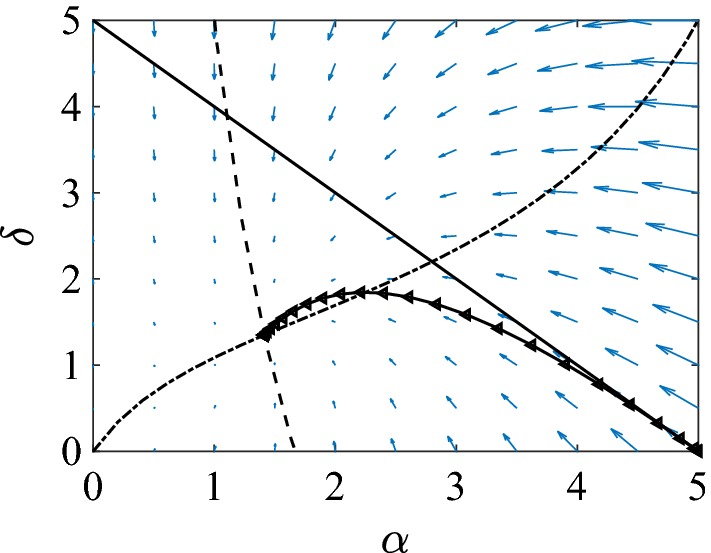
Illustration of the typical phase plane of system (4.11)–(4.12). The null clines $\delta _1$ (dashed) and $\delta _2$ (dash-dotted), and the $\alpha +\delta =n$ (continuous) line are plotted together with a typical trajectory ($\lhd$) that is attracted to the unique steady state of the system. Parameter values are $N=10{,}000$, $n=5$, $\phi =0.5$ and $\tau =\gamma =1$

The starting point is to show that system (4.11)–(4.12) admits a unique steady state which is biologically meaningful, i.e. $(\alpha ^{*},\delta ^{*}) \in D=\{(\alpha ,\delta ): 0 \le \alpha \le n, 0\le \delta \le n-\alpha \}$. First we show that the trajectories of the system remain in *D* for all appropriate initial conditions and all positive times. When $\delta =0$, then $d\delta /dt=2\tau \alpha >0$. When $\alpha =0$, then $d\alpha /dt=0$. However, by condition (4.15), $\frac{d(d\alpha /dt)}{d\alpha }=\tau [(n-1)(1-\phi )-1]>0$. Finally, we need to show that if $\alpha +\delta =n$ then $d(\alpha +\delta )/dt<0$. By substituting $\delta =n-\alpha$, and after some algebra we obtain that $d(\alpha +\delta )/dt=\gamma (\alpha -n)-\tau (n-1)\phi \alpha (1-\alpha /n)^2<0$. The observations prove that *D* is invariant. A typical picture of the phase diagram is given in Fig. 4.

It turns out that both null clines can be written conveniently with $\alpha$ being the independent and $\delta$ being the dependent variable. The null clines corresponding to $d\alpha /dt$ and $d\delta /dt$ are given by

4.13 $$\begin{aligned} \delta _{1}(\alpha )&=\frac{n}{\xi \phi }\left( \frac{\xi n (1-\phi )-1}{\alpha }+\xi \phi -1\right) , \end{aligned}$$

4.14 $$\begin{aligned} \delta _{2}(\alpha )&=\frac{2\tau \alpha }{\gamma +\tau \alpha - 2\tau \frac{\xi }{n}\phi \alpha ^2}. \end{aligned}$$

Several observations can be made. If $\xi n (1-\phi )-1>0$, then $\delta _1(\alpha )$ will be a decreasing function in $\alpha$ and its intersection with the horizontal axis is at $\alpha _1=\frac{\xi n(1-\phi )-1}{1-\xi \phi }$, which happens to be less than *n*. Furthermore, it is straightforward to show that $d\delta _{2}(\alpha )/d\alpha >0$, which means that $\delta _2(\alpha )$ is an increasing function in $\alpha$. Given the behaviour of the null clines at $\alpha =0$, it follows that their intersection gives rise to a unique steady state. Requiring that $\xi n (1-\phi )-1>0$ is equivalent to

4.15 $$\begin{aligned} \phi < \frac{n-2}{n-1}. \end{aligned}$$

This is the same as found by Keeling (1999) in the limit of $\beta =\tau n$ large and when assuming that at the threshold $C_{SI}=\gamma /\beta$. This condition can also be derived directly from Eq. (4.21) by replacing $\alpha =\tau /\gamma$ (which corresponds to the threshold $R=\frac{\tau \alpha }{\gamma }$) and then taking the limit of large $\tau$. In fact, when $\phi > (n-2)/(n-1)$ the disease dies out. Hence, the two null clines define a unique point of intersection as long as the condition above, (4.15), is met. The same argument holds even if the singularity of the second null cline happens to be in (0, *n*). However, we must also exclude the possibility that the intersection point will lie outside *D*. For example if the $\delta _2$ null cline lies to the left of $\delta _1$ then the $\delta _{2}$ null cline may cross the $\alpha +\delta =n$ boundary at a smaller value of $\alpha$ than the $\delta _{1}$ null cline does. However, this cannot happen because, in such a case, *D* would not be invariant since the solutions would leave *D* across the region of this boundary limited by the two null clines, which contradicts that fact that $d(\alpha +\delta )/dt<0$ on this boundary.

Provided that condition (4.15) holds, Fig. 4 shows that a unique steady state exists. Trapman (2007b) showed that this steady state is locally stable but global stability was not confirmed. Here, in addition to the results by Trapman (2007b) we show that under certain assumptions the existence of a limit cycle can be ruled out by applying the Bendixson criterion. This also ensures the global stability of the unique steady state. Dividing the equations by $\alpha$, the divergence of the system takes the form:

4.16 $$\begin{aligned} B(\alpha ,\delta )=\frac{d\left( \frac{d\alpha }{\alpha dt}\right) }{d\alpha }+\frac{d\left( \frac{d\delta }{\alpha dt}\right) }{d\delta }=-2\tau -\frac{\gamma }{\alpha }+\phi \left[ \frac{\tau \xi }{n}(n-\delta +2\alpha )\right] . \end{aligned}$$

We separated the above expression into the non-clustered and clustered parts of the network. It is obvious that when $\phi =0$ then $B(\alpha ,\delta )<0$ and thus no limit cycle can occur. Now setting $B(\alpha ,\delta )=0$ and neglecting the $-\gamma /\alpha$ term defines the following curve

4.17 $$\begin{aligned} \delta _{B}(\alpha )=2\alpha +n-\frac{2n}{\xi \phi }. \end{aligned}$$

This intersects the horizontal axis at $\alpha _B=\frac{n}{\xi \phi }-n/2$. Given that the slope of $\delta _{B}$ is positive, the divergence will remain negative in *D* as long as the intersection with the horizontal axis is beyond *n*. This requires that

$$\begin{aligned} \frac{n}{\xi \phi }-\frac{n}{2}>n. \end{aligned}$$

Rearranging this, we obtain

$$\begin{aligned} \phi < \frac{2n}{3(n-1)}. \end{aligned}$$

Hence, if the above holds then the unique steady state is globally stable. It is worth noting that if

$$\begin{aligned} \frac{2n}{3(n-1)}>\frac{n-2}{n-1}, \end{aligned}$$

then the global stability also holds for all $\phi <(n-2)/(n-1)$, and as long as $n< 6$ the above inequality holds.

Numerical tests suggest that global stability holds for all reasonable parameter values. For example, if the point of intersection of $\delta _{B}$ with the horizontal axis is in $(\alpha _{\beta },n)$, then the non-existence of the limit cycle can be shown as follows. To the left of $\delta _{B}$ the divergence is negative and the lower right quadrant of *D* is repellent.

#### Fast variables without clustering

When clustering is negligible and hence $\phi =0$, we find that

4.18 $$\begin{aligned} \frac{d\alpha }{dt}= & {} -\tau \alpha +\tau \xi n\alpha -\tau \alpha ^{2}, \end{aligned}$$

4.19 $$\begin{aligned} \frac{d\delta }{dt}= & {} 2\tau \alpha -\gamma \delta -\tau \alpha \delta , \end{aligned}$$

where $\xi =\frac{(n-1)}{n}$. The steady states of the system (4.18)–(4.19) are given by $(\alpha _{1}^{*},\delta _{1}^{*})=(0,0)$ and $(\alpha _{2}^{*}, \delta _{2}^{*})= \left( (n-2),\frac{2\tau (n-2)}{\gamma +\tau (n-2)}\right)$. Based on Eq. (4.10), it follows that $R=\frac{\tau \alpha _{2}^{*}}{\gamma }=\frac{\tau (n-2)}{\gamma }.$

#### Fast variables with clustering

When clustering is present in the network, the differential equations for $\alpha$ and $\delta$ are more complex and thus steady states are harder to compute. Firstly, we set Eq. (4.11) equal to zero and rearrange to isolate $\delta$, finding

4.20 $$\begin{aligned} \delta =\frac{-1+\xi n(1-\phi )+\xi \phi \alpha -\alpha }{\xi \frac{1}{n}\phi \alpha }. \end{aligned}$$

Plugging Eq. (4.20) into Eq. (4.12) leads to the following cubic equation in $\alpha$:

4.21 $$\begin{aligned}&(2\tau \xi \phi (1-\xi \phi ))\alpha ^{3}+(\tau \xi n\phi -2\tau \xi ^{2}n\phi (1-\phi )-\tau n)\alpha ^{2}\nonumber \\&\quad +(-n(\tau +\gamma )+\tau \xi n^{2}(1-\phi )+\gamma \xi n\phi )\alpha +(\gamma \xi n^{2}(1-\phi )-\gamma n)=0. \end{aligned}$$

The solution of the cubic equation (4.21) provides the steady state(s) of system (4.11)–(4.12), and allows the computation of the threshold via the formula $R^{c}=\frac{\tau \alpha ^{*}}{\gamma }$. We note that the steady state in $\alpha$ has to be biologically plausible. $\alpha =\frac{[SI]}{[I]}$ restricts the steady state to be positive and to be less than *n*, since the average number of susceptible neighbours averaged over all infected nodes needs to be less than the average degree.

### Asymptotic expansion of the epidemic threshold

The case of $\phi \ne 0$ can be regarded as a perturbation of the case without clustering and we thus set out to find $\alpha$ using a perturbation method. More precisely, we seek to find the roots of the cubic polynomial, given in Eq. (4.21), in terms of an asymptotic expansion in powers of $\phi$, that is

4.22 $$\begin{aligned} \alpha =\alpha _{0}+\phi \alpha _{1}+\phi ^{2}\alpha _{2}+\cdots . \end{aligned}$$

Plugging (4.22) into Eq. (4.21) leads to

4.23 $$\begin{aligned}&2\tau \xi \phi (1-\xi \phi )(\alpha _{0}+\phi \alpha _{1}+\phi ^{2} \alpha _{2}+\cdots )^{3} \nonumber \\&\quad +(\tau \xi n\phi -2\tau \xi ^{2}n\phi (1-\phi )-\tau n)(\alpha _{0}+\phi \alpha _{1}+\phi ^{2}\alpha _{2}+\cdots )^{2} \nonumber \\&\quad + (-n(\tau +\gamma )+\tau \xi n^{2}(1-\phi )+\gamma \xi n\phi )(\alpha _{0}+\phi \alpha _{1}+\phi ^{2}\alpha _{2}+\cdots ) \nonumber \\&\quad +(\gamma \xi n^{2}(1-\phi )-\gamma n)=0. \end{aligned}$$

Collecting terms of order $\phi ^{0}$ in (4.23) and after some algebra we find that $\alpha _{0}$ satisfies:

4.24 $$\begin{aligned} n(\alpha _0-(n-2))(\tau \alpha _0+\gamma )=0. \end{aligned}$$

Hence, $\alpha _{0}=(n-2)$. The other solution, $\alpha _0=-\gamma /\tau$ is not biologically feasible since by definition $\alpha$ is positive. As expected, $\alpha _{0}=(n-2)$ corresponds to the unclustered case. Collecting terms of order $\phi$ in (4.23), we find a polynomial in terms of $\alpha _{0}$ and $\alpha _{1}$:

4.25 $$\begin{aligned}&2\tau \xi \alpha _{0}^{3}+(\tau \xi n-2\tau \xi ^{2}n)\alpha _{0}^{2}+(\gamma \xi n-\tau \xi n^{2})\alpha _{0} \nonumber \\&\quad -2\tau n\alpha _{0}\alpha _{1}+(\tau \xi n^{2}-n(\tau +\gamma ))\alpha _{1}-\gamma \xi n^{2}=0. \end{aligned}$$

Equation (4.25) leads to

$$\begin{aligned} \alpha _{1}=\frac{\gamma \xi n^{2}-2\tau \xi \alpha _{0}^{3}+(2\tau \xi ^{2}n-\tau \xi n)\alpha _{0}^{2}+(\tau \xi n^{2}-\gamma \xi n)\alpha _{0}}{\tau \xi n^{2}-n(\tau +\gamma )-2\tau n\alpha _{0}}, \end{aligned}$$

which, after substituting $\alpha _{0}=(n-2)$ and $\xi =\frac{(n-1)}{n}$ yields

4.26 $$\begin{aligned} \alpha _{1}=\frac{-2(n-1)}{n^{2}}\left( \frac{2\tau (n-1)(n-2)+\gamma n}{\tau (n-2)+\gamma }\right) . \end{aligned}$$

To summarise, we have determined the first two coefficients $\alpha _{0}$ and $\alpha _{1}$ of the asymptotic expansion (4.22) which solves the cubic equation (4.21). Hence, the true solution is approximated by:

4.27 $$\begin{aligned} \alpha =(n-2)-\phi \frac{2(n-1)}{n^{2}}\left( \frac{2\tau (n-1)(n-2)+\gamma n}{\tau (n-2)+\gamma }\right) +{\mathcal {O}}(\phi ^{2}). \end{aligned}$$

We make several remarks. First, the epidemic threshold will be given by $R^{c}=\tau \alpha /\gamma$. Second, the coefficient of the first order correction of $\alpha$ can be rearranged in terms of $R=\frac{\tau (n-2)}{\gamma }$, the threshold for the case of unclustered networks, leading to

4.28 $$\begin{aligned} R^{c}=R-\phi a\frac{\tau }{\gamma }\left( \frac{aR+1}{R+1}\right) , \end{aligned}$$

where $a=2(n-1)/n$ and where terms in $\phi$ of order larger than one have been neglected.

Finally, it is clear that due to the first order correction being negative, we have that

4.29 $$\begin{aligned} R^{c}=R-\phi a\frac{\tau }{\gamma }\left( \frac{aR+1}{R+1}\right) \le R=\frac{\tau (n-2)}{\gamma }. \end{aligned}$$

**Fig. 5:**
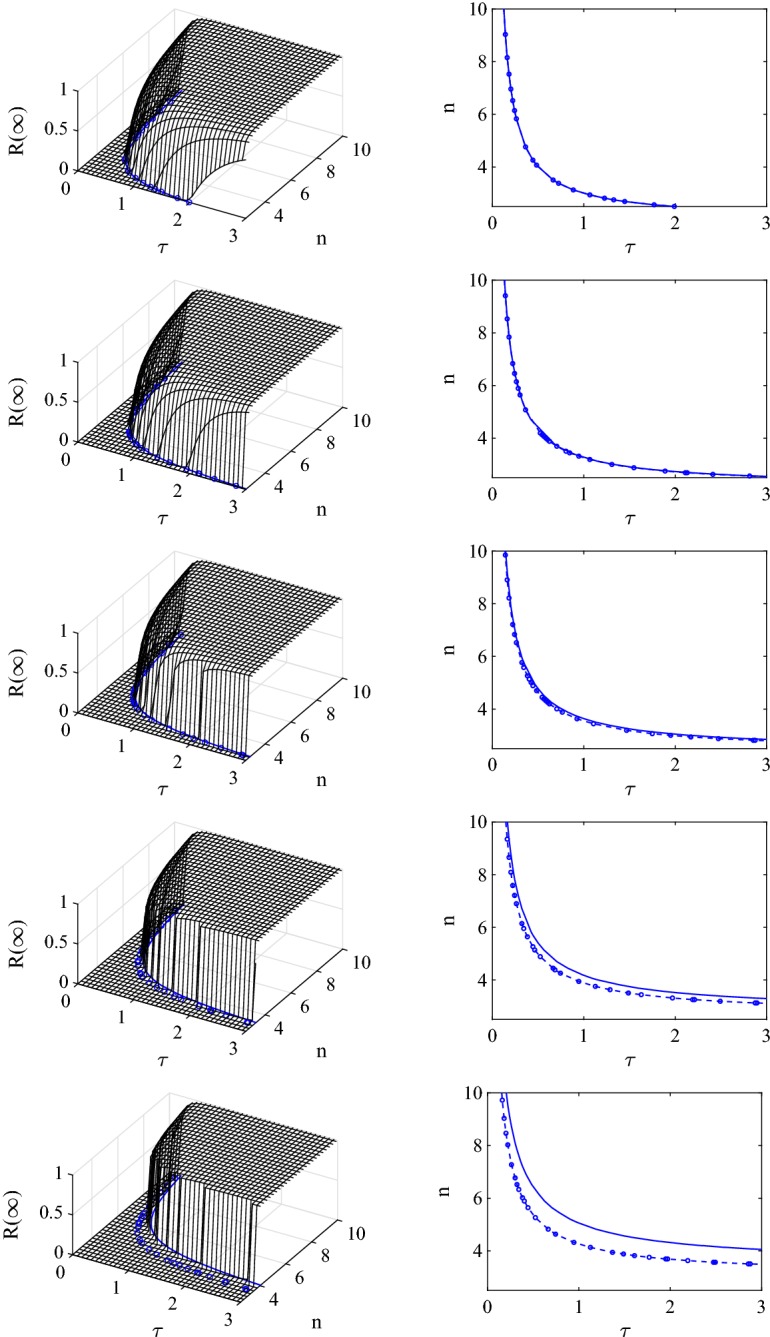
Assessing the validity of the epidemic threshold based on the asymptotic approximation (4.27) (dashed line and markers—$\circ$) by comparing it to the epidemic threshold based on the numerical solution of the cubic equation (4.21) (continuous lines). In the right hand column we compare both threshold curves in the $(\tau ,n,0)$ plane. In the left hand column both curves are compared to the final epidemic size based on numerical integration of the pairwise model equations with the simple closure. Parameter values are $N=10{,}000$, $\gamma =1$ and from top to bottom the clustering coefficients are $\phi =0, 0.15, 0.3, 0.45, 0.6$

The goodness of the estimate for $\alpha$ (4.27) is tested by comparing it to the numerical solution of the cubic equation (4.21). This is done in Fig. 5 for five different values of the clustering coefficient. The asymptotic approximation performs well and only breaks down for values of clustering larger than $\phi \simeq 0.3$. From the same figure it is clear that higher values of clustering push the critical $R^{c}=1$ curve to higher values of $\tau$ and *n*. Hence, in the presence of clustering a viable epidemic requires either a denser network or a higher transmission rate, noting that the transmission rate and the recovery rate $\gamma$ are not strictly independent.

### Numerical examples

In the previous section we have demonstrated that for the pairwise model with the simplest closure for clustered networks, the determination of the epidemic threshold involves the solution of a cubic equation. While this solution can be obtained numerically, we presented an asymptotic approximation of the solution in terms of powers of the clustering coefficient $\phi$. In Fig. 5 we present a systematic test of the newly determined threshold by comparing the threshold based on the numerical solution of the cubic equation (4.21) (continuous line in the ($\tau ,n,0$) plane), the asymptotic approximation of the solution to the cubic equation (4.27) (dashed line and markers—$\circ$) and the numerical solution of the full ODE system corresponding to the closed pairwise model (4.1)–(4.5).

The agreement between the explicit numerical solution of the closed pairwise system and threshold based on the numerical solution of the cubic equation is excellent for all clustering values and other parameter combinations. Moreover, the agreement of these results with the threshold based on the asymptotic approximation is also excellent and remains valid for values of $0\le \phi \le 0.3$. The initial conditions for the closed pairwise systems were set in the following way: $[I](0)=I_0=1$, $[S](0)=N-I_0=S_0$, $[SI](0)=nI_0\frac{S_0}{N}$, $[SS](0)=nS_0\frac{S_0}{N}$ and $[II](0)=nI_0\frac{I_0}{N}$. The ODEs were run for a sufficiently long time ($T_{max}=1000$) to ensure that the epidemic died out. It is worth noting that the correct numerical solution of the cubic equation can be chosen by keeping in mind that $0\le \alpha =\frac{[SI]}{[I]}\le n$.

## Results for the pairwise model with the compact improved closure

Starting from the improved closure (3.14) but in line with Proposition 2, we adapt the closure so that the term responsible for the approximation on the clustered part of the network does not consider variables, singles or pairs involving the R class. This leads to the new closure

5.1 $$\begin{aligned}{}[ASI]=(n-1)\left( (1-\phi )\frac{[AS][SI]}{n[S]}+\phi \frac{[AS][SI][IA]}{[A]\left( \frac{[SS][SI]}{[S]}+\frac{[SI][II]}{[I]}\right) }\right) , \end{aligned}$$

which we refer to as the compact improved closure. Plugging Eq. (5.1) into the exact system (2.2)–(2.6) leads to a self-consistent system that is written out in full in Appendix A.

In line with our procedure so far, we aim to find the epidemic threshold of this new pairwise system with the compact improved closure. It turns out that the approach used for the pairwise system with the simple closure is applicable to this case, and the steps and results are summarised below.

### Fast variables with the compact improved closure

As we have shown before, finding the threshold relies on finding the quasi-equilibrium of $\alpha =\frac{[SI]}{[I]}$. In Appendix A we show that this requires knowledge about the behaviour of $\delta =\frac{[II]}{[I]}$ variable and indeed a system of differential equations involving these two variables can be derived. This system is given below

5.2 $$\begin{aligned} \frac{d\alpha }{dt}= & {} -\tau \alpha -\tau \alpha ^{2}+\tau (n-1) \left( (1-\phi )\alpha +\phi \alpha \left( \frac{n-\delta }{n+\delta }\right) \right) , \end{aligned}$$

5.3 $$\begin{aligned} \frac{d\delta }{dt}= & {} 2\tau \alpha -\gamma \delta +2\tau (n-1) \left( \frac{\phi \alpha \delta }{n+\delta }\right) -\tau \alpha \delta . \end{aligned}$$

As previously, the steady states of this system are of interest and apart from the trivial $(\alpha ^{*},\delta ^{*})=(0,0)$ steady state, the quasi-equilibrium can be found by first expressing $\delta$ as a function of $\alpha$. This can be done by setting Eq. (5.2) equal to zero and rearranging, leading to

5.4 $$\begin{aligned} \alpha =(n-2)-(n-1)\phi \frac{2\delta }{n+\delta }. \end{aligned}$$

Plugging Eq. (5.4) into Eq. (5.3) and collecting powers of $\delta$ leads to the following cubic equation

5.5 $$\begin{aligned}&(-A-B)\delta ^{3}+(-n(n-2)-A^2-2nB)\delta ^{2}\nonumber \\&\quad +(-n(n-2)A+2nA-n^2B)\delta +2n^2(n-2)=0, \end{aligned}$$

where $A=(n-2)-2\phi (n-1)$ and $B=\gamma /\tau$. It is worth noting that in this case it is easier to work with $\delta$, but any results can be converted in terms of $\alpha$ which is the main variable of interest. However, before we proceed with the asymptotic expansion of the solution, we show that there is a unique biologically feasible steady state.

### Global stability of the steady state

It is worth considering whether the trajectories of the system governed by Eqs. (5.2)–(5.3) remain in $D=\{(\alpha ,\delta ): 0 \le \alpha \le n, 0\le \delta \le n-\alpha \}$ for all appropriate initial conditions and all positive times. When $\alpha =0$, then $d\alpha /dt=0$, so the $\alpha =0$ line is stationary and solutions remain in *D*. Moreover, on this line, $\frac{d(d\alpha /dt)}{d\alpha }=\frac{\tau n(n-2)}{n+\delta }+\frac{\delta \tau ((n-2)-2\phi (n-1))}{n+\delta }$ which is greater than zero when $2\phi <(n-2)/(n-1)$. This is a condition which will resurface later when the intersection of the null clines is analysed. If $\delta =0$, then $d\delta /dt=2\tau \alpha >0$ meaning that the solution cannot leave *D* along the $\delta =0$ line. Finally, we need to show that if $\alpha +\delta =n$ then $d(\alpha +\delta )/dt<0$. By substituting $\delta =n-\alpha$, and after some algebra we obtain that $d(\alpha +\delta )/dt=-\gamma (n-\alpha )=-\gamma \delta <0$. These findings prove that *D* is invariant.

To continue we focus on showing that (5.2)–(5.3) admits a unique steady state which is biologically meaningful, i.e. $(\alpha ^{*},\delta ^{*}) \in D$. The null cline corresponding to $d\alpha /dt$ can be rewritten to give

5.6 $$\begin{aligned} \delta _n(\alpha )=\frac{n((n-2)-\alpha )}{\alpha +2\phi (n-1)-(n-2)}. \end{aligned}$$

It is straightforward to check that

**Fig. 6:**
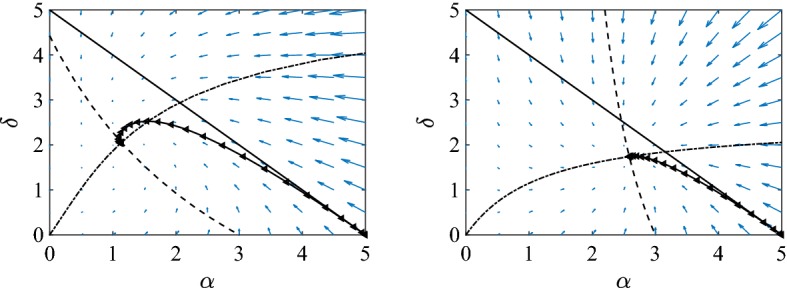
Illustration of the typical phase plane of system (5.2)–(5.3). The null clines $\delta _n$ (dashed) and $\alpha _n$ (dash-dotted), and the $\alpha +\delta =n$ (continuous) line are plotted together with a typical trajectory ($\lhd$) that is attracted to the unique steady state of the system. Parameter values are $N=10{,}000$, $n=5$, $\phi =0.8$ (left panel), $\phi =0.2$ (right panel) and $\tau =\gamma =1$

5.7 $$\begin{aligned} d\delta _n(\alpha )/d\alpha =\frac{-2\phi n(n-1)}{(\alpha +2\phi (n-1)-(n-2))^2}<0, \end{aligned}$$

meaning that the function is decreasing for all $\alpha$. Setting $\alpha =0$ in (5.6) leads to $\delta =n(n-2)/(2\phi (n-1)-(n-2))$, which can be both negative or positive. On the other hand setting $\delta =0$ in (5.6) yields $\alpha =(n-2)$. This null cline has a singularity at $\alpha ^{*}=(n-2)-2\phi (n-1)$, with $\alpha ^{*}<(n-2)<n$. If $\alpha ^{*}<0$ then the branch on the left of the vertical asymptote will not intersect *D*. This happens exactly when $2\phi > (n-2)/(n-1)$. So in this case the branch of the null cline to the right of the asymptote intersects the $\alpha$-axis at $((n-2),0)$ and the $\delta$-axis at $(0,n(n-2)/(2\phi (n-1)-(n-2)))$, where the intersection with the $\delta$-axis happens at a positive value, namely $n(n-2)/(2\phi (n-1)-(n-2))>0$, and this inequality holds true due to requiring that $\alpha ^{*}$ is negative. This point may be greater than *n* but also intersects the horizontal axis at $(n-2,0)$. This is illustrated in Fig. 6 (left panel). When the singularity point is positive, $\alpha ^{*}>0$, that is when $2\phi < (n-2)/(n-1)$, then the intersection with the $\delta$-axis happens at a negative value of $\delta$. This is also illustrated in Fig. 6 (right panel), where the positive singularity is clearly visible with the intersection with the $\delta$-axis being out of the range of the plot.

The null cline corresponding to $d\delta /dt$ is given by

5.8 $$\begin{aligned} \alpha _n(\delta )=\frac{\gamma \delta (n+\delta )}{\tau \{- \delta ^2+[2(n-1)\phi - (n-2)]\delta +2n\}}. \end{aligned}$$

This null cline passes through $(\alpha ,\delta )=(0,0)$ and the derivative of $\alpha _n(\delta )$ is always positive, namely,

5.9 $$\begin{aligned} d\alpha _n(\delta )/d\delta =\frac{\gamma (2\delta ^2+2n^2+4n\delta +2\phi \delta ^2(n-1))}{\tau \{- \delta ^2+[2(n-1)\phi - (n-2)]\delta +2n\}^2}\ge 0. \end{aligned}$$

The denominator is a quadratic polynomial in $\delta$ with the discriminant being always positive and thus leading to two distinct real roots. From the equation it follows that sum of the roots is $(n-2)-2\phi (n-1)$ and their product is $-2n<0$. Therefore, two singularity points exist, one for negative and the other for positive $\delta$. $\alpha _n(\delta )$ is such that

$$\begin{aligned} \lim _{\delta \rightarrow \pm \infty }\alpha _n(\delta )=-\gamma /\tau . \end{aligned}$$

Hence, *D* happens to lie, at least partly, in the area defined by the two singularity points (i.e. the region between the two vertical asymptotes if considered in the $(\delta ,\alpha )$ plane). In this area the null cline increases with $\delta$ starting from $(\alpha ,\delta )=(0,0)$, see both panels in Fig. 6. Summarising, we have shown that the null clines will intersect at a unique point, and this point cannot be outside *D* due to the orientation of the vector fields, see also the argument presented in Sect. 4.3.1.

Finally, we show that the existence of a limit cycle can be ruled out by applying the Bendixson criterion. This also ensures the global stability of the unique steady state. Dividing Eqs. (5.2)–(5.3), and computing $B(\alpha ,\delta )=\frac{d}{d\alpha }\left( \frac{1}{\alpha } \frac{d\alpha }{dt}\right) +\frac{d}{d\delta }\left( \frac{1}{\alpha } \frac{d\delta }{dt}\right)$, the divergence of the system yields

5.10 $$\begin{aligned} B(\alpha ,\delta )=-2\tau -\frac{\gamma }{\alpha }+\frac{2\tau \phi n (n-1)}{(n+\delta )^2}. \end{aligned}$$

It is easy to show that this is negative. Even if $-\frac{\gamma }{\alpha }$ is neglected, we have that

$$\begin{aligned} -2\tau +\frac{2\tau \phi n (n-1)}{(n+\delta )^2}=-2\tau \frac{(n+\delta )^2-\phi n(n-1)}{(n+\delta )^2}<0, \end{aligned}$$

since $(n+\delta )$ is greater than both *n* and $(n-1)$.

### Asymptotic expansion of the epidemic threshold

As in Sect. 4.4, we require the roots of the cubic polynomial given in Eq. (5.5). To do so, we express $\delta$ as an asymptotic expansion in powers of $\phi$. We substitute

5.11 $$\begin{aligned} \delta =\delta _{0}+\delta _{1}\phi +\delta _{2}\phi ^{2}+\cdots . \end{aligned}$$

Plugging the expansion for $\delta$ (5.11) into Eq. (5.5) leads to

5.12 $$\begin{aligned}&(-A-B)(\delta _{0}+\delta _{1}\phi +\delta _{2}\phi ^{2}+\cdots )^{3} \nonumber \\&\quad +(-n(n-2)-A^{2}-2nB)(\delta _{0}+\delta _{1}\phi +\delta _{2}\phi ^{2}+ \cdots )^{2} \nonumber \\&\quad +(-n(n-2)A+2nA-n^{2}B)(\delta _{0}+\delta _{1}\phi +\delta _{2} \phi ^{2}+\cdots )+2n^{2}(n-2)=0. \end{aligned}$$

Alternatively, substituting (5.4) into the differential equation for $\delta$ (5.3), setting the expression equal to zero and rearranging leads to

5.13 $$\begin{aligned} \gamma \delta (n+\delta )^{2}= & {} \tau [(n-2)(n+\delta )-2\phi (n-1) \delta ] \nonumber \\&[(2-\delta )(n+\delta )+2\phi (n-1)\delta ]. \end{aligned}$$

Substituting (5.11) into (5.13) and collecting terms of order $\phi ^{0}$ yields

5.14 $$\begin{aligned} \gamma \delta _{0}(n+\delta _{0})^{2}= & {} \tau [(n-2)(n+\delta _{0})] [(2-\delta _{0})(n+\delta _{0})] \end{aligned}$$

5.15 $$\begin{aligned} \gamma \delta _{0}= & {} \tau (n-2)(2-\delta _{0}) \end{aligned}$$

5.16 $$\begin{aligned} \delta _{0}(\gamma +\tau (n-2))= & {} 2\tau (n-2) \end{aligned}$$

5.17 $$\begin{aligned} \delta _{0}= & {} \frac{2\tau (n-2)}{\gamma +\tau (n-2)}. \end{aligned}$$

Following the same process to collect terms of order $\phi ^{1}$, we find

5.18 $$\begin{aligned} \gamma \delta _{1}[(n+\delta _{0})^{2}+2(n+\delta _{0})\delta _{0}]&=\tau (n-2)(n+\delta _{0})[\delta _{1}(2-n-2\delta _{0})+2(n-1)\delta _{0}] \nonumber \\&\quad +\tau (2-\delta _{0})(n+\delta _{0})[(n-2)\delta _{1}-2(n-1)\delta _{0}], \end{aligned}$$

which can be rearranged to yield

5.19 $$\begin{aligned} \delta _{1}=\frac{2\tau (n-1)\delta _{0}(n-4+\delta _{0})}{\gamma (n+3\delta _{0})+\tau (n-2)(n+3\delta _{0}-4)}, \end{aligned}$$

with $\delta _{0}$ defined in (5.17). In summary, we have determined the first two coefficients $\delta _{0}$ and $\delta _{1}$ of the asymptotic expansion for $\delta$ given in Eq. (5.11). Hence, the true solution is approximated by the following expression:

5.20 $$\begin{aligned} \delta =\frac{2\tau (n-2)}{\gamma +\tau (n-2)}+\frac{2\tau (n-1) \delta _{0}(n-4+\delta _{0})\phi }{\gamma (n+3\delta _{0})+\tau (n-2) (n+3\delta _{0}-4)}+{\mathcal {O}}(\phi ^{2}). \end{aligned}$$

Finally, we are able to plug (5.20) into the quasi-equilibrium point for $\alpha$, given in Eq. (5.4), to obtain

5.21 $$\begin{aligned} \alpha =(n-2)-2(n-1)\phi \frac{\delta _{0}}{n+\delta _{0}}+{\mathcal {O}}(\phi ^{2}), \end{aligned}$$

which, upon neglecting terms in $\phi$ of order larger than one, can be rearranged to find

5.22 $$\begin{aligned} \alpha =(n-2)-\phi \frac{4\tau (n-1)(n-2)}{\tau (n+2)(n-2)+\gamma n}. \end{aligned}$$

The expression for $\alpha$ (5.22) can be used to determine the epidemic threshold as follows

5.23 $$\begin{aligned} R^{cci}=\frac{\tau \alpha }{\gamma }=\frac{(n-2)\tau }{\gamma }- \phi \frac{\tau }{\gamma }\left( \frac{4\tau (n-1)(n-2)}{\tau (n+2)(n-2)+\gamma n}\right) . \end{aligned}$$

It is straightforward to see that again $R^{cci}\le R$, with clustering making the spread of the epidemic less likely.

### Numerical examples

**Fig. 7:**
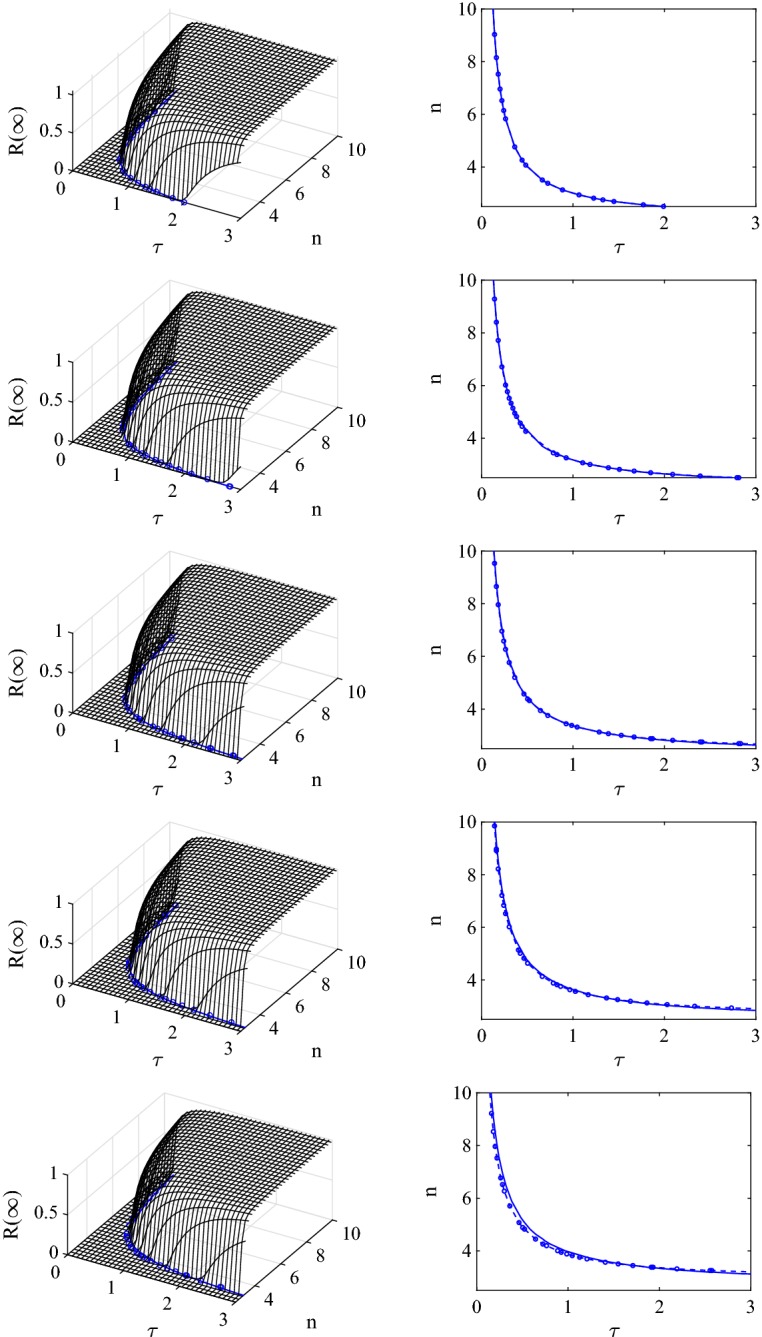
Assessing the validity of the epidemic threshold based on the asymptotic expansion (5.20) (dashed line and markers—$\circ$) by comparing it to the epidemic threshold based on the numerical solution of the cubic equation (5.5) (continuous lines). In the right hand column we compare both threshold curves in the $(\tau ,n,0)$ plane. In the left hand column both curves are compared to the final epidemic size based on numerical integration of the pairwise model equations with the compact improved closure. Parameter values are $N=10{,}000$, $\gamma =1$ and from top to bottom the clustering coefficients are $\phi =0, 0.15, 0.3, 0.45, 0.6$

In Fig. 7 we repeat the systematic test of comparing the epidemic threshold generated via the numerical solution of the cubic equation (5.5), the epidemic threshold generated by the asymptotic expansion (5.23) and the numerical value of the final epidemic size predicted by the pairwise model with the compact improved closure, over a wide range of $(\tau ,n)$ values. Several observations can be made. First, it is clear that higher values of clustering push the location of the threshold to higher $\tau$ and *n* values, meaning that the limiting effect of clustering on the epidemic spread can only be overcome if either the value of the transmission rate or average degree increases. Second, the agreement between the threshold based on the numerical solution of the cubic equation (5.5) and the asymptotic expansion (5.20) is excellent over a wide range of $\phi$ values. In fact, in this case the agreement is excellent for $0\le \phi \le 0.45$, with only small deviations even for $\phi =0.6$. The agreement between the numerical solution of the pairwise model and the threshold based on the numerical solution of the cubic equation (5.5) remains excellent across all parameter values.

## Comparing epidemic thresholds based on different models

Exploiting the presence of fast variables and combining this with elements of perturbation theory allowed us to compute the epidemic threshold for the pairwise model with two different closures that take clustering into account. Our results are in line with the findings by Li et al. (2018) and Miller (2009b). Li et al. (2018) calculated the epidemic threshold in a pairwise model for clustered networks with a closure based on the number of links in a motif, rather than nodes. This led to

6.1 $$\begin{aligned} R_0=\frac{(n-1)\tau }{\tau +\gamma +\tau \phi }. \end{aligned}$$

Equation (6.1) can be expanded in terms of $\phi$ to give

6.2 $$\begin{aligned} R_0=\frac{(n-1)\tau }{\tau +\gamma }\left( \frac{1}{1+\phi \frac{\tau }{\tau +\gamma }}\right) \simeq \frac{(n-1)\tau }{\tau +\gamma }\left( 1-\phi \frac{\tau }{\tau +\gamma }+\cdots \right) , \end{aligned}$$

which again reflects our finding that clustering reduces the epidemic threshold.

Similarly but for clustered networks with heterogeneous degree distributions, Miller (2009b) found that

6.3 $$\begin{aligned} R_0=\frac{\langle k^2-k\rangle }{\langle k\rangle }T-\frac{2\langle n_{\triangle }\rangle }{\langle k\rangle }T^2+\cdots , \end{aligned}$$

where $\langle k^i \rangle$ stands for the *i*th moment of the degree distribution, *T* is the probability of infection spreading across a link connecting an infected to a susceptible node and $\langle n_{\triangle }\rangle$ denotes the average number of triangles that a node belongs to. The expression above again shows that clustering reduces the epidemic threshold when compared to the unclustered case. Furthermore, if the network is regular and we assume that infections and recoveries are Markovian processes with rates $\tau$ and $\gamma$ respectively, giving $T=\tau /(\tau +\gamma )$, $R_0$ above reduces to

6.4 $$\begin{aligned} R_0=\frac{\tau (n-1)}{\tau +\gamma }-(n-1)\phi \left( \frac{\tau }{\tau +\gamma }\right) ^2+\cdots , \end{aligned}$$

where we have used the fact that a global clustering coefficient of $\phi$ translates to a node on average being part of $\frac{1}{2}n(n-1)\phi$ uniquely counted triangles. This in turn coincides with Eq. (6.2). This is perhaps unexpected since the first expression was obtained based on a new type of closure for pairwise models while the other expression was based on percolation theory type arguments. Trapman (2007a) considered specific networks with household structure to investigate the effects of clustering and infectious period distribution on a modified version of $R_0$ referred to as $R_{*}$, and lower and upper bounds for the value of this quantity were found. Similarly Ball et al. (2010) considered a random network incorporating household structure and provided the basic reproduction number which takes into account within household and global contacts.

However, as elaborated upon in Sect. 4.1, the *R* threshold that we compute is a growth-rate-based threshold and whilst at the threshold $R=1 \Longleftrightarrow R_0=1$, *R* does not have the same biological interpretation as $R_0$. Despite this, our analysis confirms that clustering starves the spreading epidemic of susceptible neighbours such that the epidemic is less likely to spread if the networks are clustered, all other parameters being equal. More importantly, the epidemic threshold is model-dependent and the pairwise model with the compact improved closure leads more readily to epidemic outbreaks when compared to the pairwise model with the simple closure, see Figs. 5 and 7. While this ordering is true for the parameters used in this paper, we cannot conclude that this ordering holds true for all parameter values. Further research may focus on the ordering of these thresholds and gaining a better understanding of the impact of model choice on the values of the epidemic threshold.

The computation of the true $R_0$ for pairwise models can be attempted by considering the next generation matrix approach (Van den Driessche and Watmough 2002). Looking at the pairwise model with the simplest closure and ordering the variables involved in the spreading process as: [*I*],[*SI*], the generation of new infectious cases at the the disease-free steady state is given by

6.5 $$\begin{aligned} F = \begin{pmatrix} 0 &{}\quad \tau \\ 0 &{}\quad \tau (n-1)(1-\phi )+\tau \xi \phi \alpha \end{pmatrix}, \end{aligned}$$

where the lower right term is obtained from Eq. (4.3) by looking at the rate of growth of [*SI*] in terms of [*SI*] itself and evaluating it at the disease-free equilibrium, that is

$$\begin{aligned} \dot{[SI]}=+\tau \xi \frac{[SS]}{[S]}\left( (1-\phi )+\phi \frac{N}{n} \frac{[SI]}{[S][I]}\right) [SI]\simeq \left( \tau (n-1)(1-\phi )+\tau \xi \phi \alpha \right) [SI]. \end{aligned}$$

Now all other transfers between compartments are summarised in the *V* matrix, which is given below

6.6 $$\begin{aligned} V = \begin{pmatrix} \gamma &{}\quad 0 \\ 0 &{}\quad (\tau +\gamma )+\tau \frac{\xi }{n}\alpha \delta \phi \end{pmatrix}, \end{aligned}$$

where the lower right term describes the rate at which [*SI*] pairs are depleted. This is obtained from Eq. (4.3) as follows

$$\begin{aligned} \dot{[SI]}= & {} -\left( (\tau +\gamma )+\tau \xi \frac{[SI]}{[S]}(1-\phi )+ \phi \tau \xi \frac{[SI]}{[S]}\frac{N[II]}{n[I]^{2}}\right) [SI] \\\simeq & {} -((\tau +\gamma )+\tau \frac{\xi }{n}\alpha \delta \phi )[SI], \end{aligned}$$

where again all expressions were evaluated at the disease free steady state. Now $R_0$ is given by the leading eigenvalue of $FV^{-1}$, which is

6.7 $$\begin{aligned} R_0=\frac{\tau n (n-1)-\tau (n-1)(n-\alpha )\phi }{n(\tau +\gamma )+\tau \xi \alpha \delta \phi }. \end{aligned}$$

Obviously, this seems like a rather complicated expression since the quasi-equilibrium values for $\alpha$ and $\delta$ are needed. These are only available as asymptotic expansions in powers of $\phi$. Nevertheless, for $\phi =0$, $R_0=\frac{\tau (n-1)}{\tau +\gamma }$, which agrees perfectly with the two results quoted above. Considering the $\phi >0$ case, we write $R_0=r_0+\phi r_1$, $\alpha =\alpha _0+\phi \alpha _1$ and $\delta =\delta _0+\phi \delta _1$. Plugging these into Eq. (6.7), leads to

$$\begin{aligned} r_0=\frac{\tau (n-1)}{\tau +\gamma }\,\,\,\text {and}\,\,\, r_1=-\frac{\tau ^2(n-1)}{(\tau +\gamma )^2}\left[ \frac{2(\tau +\gamma )}{n\tau } +\frac{(n-1)}{n}\alpha _0\delta _0\right] . \end{aligned}$$

While the first term in the expansion for $R_0$ agrees with the results quoted above, the second term seems less likely to be equivalent to those shown above. This same approach can be used to compute $R_0$ when the compact improved closure is used. We believe that comparing these different ways of computing the epidemic threshold can contribute to reconciling different methods and will lead to more clarity and transparency between various modelling approaches.

Finally, we report some results concerning networks composed of two layers, local within household and global contacts, where epidemic threshold-like quantities have been proposed (Ball et al. 2010). Taking the infection rates over global/network and local/household edges to be the same means that households in the model can be viewed as a device for introducing clustering into the network. This observation motivates our short analysis below. We consider the simple example of a network with all households of size three with additional global contacts assigned to nodes according to a configuration-like network with a regular degree, say $\mu _D$. This is to keep in line with our assumption of regular random networks. Based on results by Ball et al. (2010), the clustering in such a network is

6.8 $$\begin{aligned} \phi =\frac{2}{2+\mu _D(3+\mu _D)}, \end{aligned}$$

which can be inverted to give $\mu _D$ in terms of clustering

6.9 $$\begin{aligned} \mu _D=\frac{1}{2}\sqrt{1+\frac{8}{\phi }}-\frac{3}{2}. \end{aligned}$$

Assuming that both infection and recovery are Markovian with rates $\lambda _G$ (infection through global links), $\lambda _L$ (infection within households) and $\gamma$, and following the calculations by Ball et al. (2010) it is easy to show that the epidemic threshold is

6.10 $$\begin{aligned} R_{*}= & {} (1-M(\lambda _{G}))((1+\mu _{T})\mu _D-1) \nonumber \\= & {} -(1-M(\lambda _{G}))+ (1-M(\lambda _{G}))(1+\mu _{T})\mu _D, \end{aligned}$$

where

6.11 $$\begin{aligned} M(\theta )=\frac{\theta }{\gamma +\theta },\quad \mu _T=2\left[ 1-M^2(\lambda _L)-M(2\lambda _L)(1-M(\lambda _L)\right] . \end{aligned}$$

Plugging in the expression for $\mu _D$, as in Eq. (6.9), leads to

6.12 $$\begin{aligned} R_{*}= & {} \underbrace{-(1-M(\lambda _G))-\frac{3}{2}(1-M(\lambda _{G})) (1+\mu _{T})}_{T_1} \nonumber \\&+\underbrace{\frac{1}{2}(1-M(\lambda _G))(1-\mu _T)}_{T_2} \sqrt{1+\frac{8}{\phi }} \end{aligned}$$

It is now obvious that $R_{*}$ decreases as $\phi$ increases, but to keep in the spirit of this section we expand the above in terms of $\phi$. Given that around $x=0$ the following expansion holds $\sqrt{1+8/x}=\sqrt{\frac{1}{x}}\left( 2\sqrt{2}+\frac{1}{4\sqrt{2}}x-\cdots \right)$, we can rewrite $R_{*}$ to give

6.13 $$\begin{aligned} R_{*}=T_1+2\sqrt{2}T_2\frac{1}{\sqrt{\phi }}+\frac{1}{4\sqrt{2}}T_2\sqrt{\phi }-\cdots . \end{aligned}$$

Two important remarks can be made. First, even though $R_{*}$ defines an epidemic threshold, it does not have the same interpretation as the basic reproduction number: it is the household reproduction number. However, it is a threshold parameter so it takes a value below/at/above its threshold value ($=1$) precisely when any other threshold parameter (such as $R_0$) is below/at/above its threshold value. Secondly, the dependency on $\phi$ for the various epidemic thresholds differs. While for most thresholds considered here this dependency is via a negative term of ${\mathcal {O}}(\phi )$, the threshold from the household model decreases as ${\mathcal {O}}((\phi )^{-1/2})$ as $\phi$ increases away from zero. This may indicate a clear difference in the underlying models but all models may be correct as long as their individual assumptions are met. Therefore, further exploration may focus on understanding which assumptions lead to this discrepancy and what the implications of the various modelling approaches are when applying such models in reality.

## Discussion

In this paper we derived an analytic epidemic threshold using pairwise models but for clustered networks. For the unclustered case this problem has been solved previously (Keeling 1999). Here, however, by exploiting the presence of fast variables and using elements of perturbation theory, we were able to find the epidemic threshold as an asymptotic expansion in powers of the clustering coefficient.

Our analysis confirms that clustering starves the spreading epidemic of susceptible nodes such that the epidemic is less likely to spread if the networks are clustered, all other parameters being equal. More importantly, the epidemic threshold is model-dependent and the pairwise model with the compact improved closure leads more readily to epidemic outbreaks when compared to the pairwise model with the simple closure, see Figs. 5 and 7. While this ordering is true for the parameters used in this paper, it is easy to show that this relation can change if parameters are tuned accordingly.

We carried out a full analysis of two systems of fast variables (one corresponding to the simplest closure with no clustering, the other corresponding to the compact improved closure for clustered networks). Both systems exhibit similar behaviours but, surprisingly, the more complicated one (that with the compact improved closure) yields results with virtually no constraints on the parameter values.

It is obvious that the complexity of the closure has a bearing on the complexity of the resulting model. As shown in the paper, using the compact improved closure leads to a more complex model whose analysis is slightly more complicated. After submitting the present paper and while waiting for the reviews, we analysed the system with the full improved closure (Kiss et al. 2018). However, our analysis only included the asymptotic expansion of the epidemic threshold without considering the detailed analysis of the system of fast variables (e.g. existence and uniqueness of a biologically feasible steady-state). This system is now four dimensional with not two but four fast variables (the extra variables being [*SR*] / [*R*] and [*IR*] / [*I*]). In doing so, we were able to confirm the effectiveness and generality of the approach presented in the paper.

It will also be worthwhile to compare different models in order to identify the impact of clustering on epidemics by mapping out regions in the parameter space where its effect is strongest. It is known that when the network is dense the effect of clustering is limited and the same holds when the transmission/recovery rates are high/low, respectively. Moreover, as we have shown in Sect. 6 there is scope for reconciling epidemic thresholds computed from different mean-field or stochastic models where the network is a key ingredient. More importantly, while there is some agreement between the different epidemic threshold expressions, especially in some limits or particular cases, it is clear that the epidemic threshold is model dependent. Hence, the biology of the disease and the contact pattern has to be carefully analysed and taken into account when choosing models that are to be used in relation to actual epidemics.

Of course there remains the issue of accounting for degree heterogeneity in the network and this has been addressed to some extent by using percolation type approaches. The approach that we presented in this paper may be extended to degree-heterogeneous clustered networks, but this will require more sophisticated models such as effective-degree, or compact/super-compact pairwise models (Simon and Kiss 2015). These will no doubt lead to more complex systems which are more challenging to analyse. The simplest starting point could be to consider a network with nodes having either degree $k_1$ or $k_2$. For ease of treatment, let $N_i$ be the number of nodes with degree $k_i$ with $i \in \{1,2 \}$. Now one can assume that clustering in the network is introduced at random so it is going to be proportional to the degree and the mixing between the two groups of nodes. One can assume the simplest case of proportional mixing, where the number of links between nodes of degree $k_i$ and $k_j$, $n_{i,j}$ is simply $n_{ij}=\frac{k_i k_j N_i N_j}{\sum _l k_l N_l}$. Then, the closure could be considered as follows

7.1 $$\begin{aligned}{}[ASI]&=(1-\phi )[ASI]+\phi [ASI]=(1-\phi )\sum _{i}[AS_{i}I]+\phi \sum _{i}[AS_{i}I], \end{aligned}$$

where $S_{i}$ denotes the class of susceptible nodes of degree $k_i$. Now appropriately scaled closures for the triples are needed, which will depend on the degree of the nodes and how clustering is apportioned over nodes of different degrees. The viability of such a model will then rely on whether such closures are compact and compatible enough to derive a reasonably simple overall expression for [*ASI*], ideally one where the closure no longer depends on degree, but rather such information appears as some factor in the closure.

Finally, it would be worthwhile to test our findings against explicit stochastic network simulations. Since our focus was on exploiting the presence of fast variables and the use of perturbation analysis to determine the epidemic threshold analytically, such empirical validation was thought to be beyond the scope of the present work. We hope that the results of this paper may encourage other researchers to consider and tackle the challenges posed by modelling epidemic dynamics on clustered networks with heterogeneous degree distributions.

## Derivation of evolution equations for the fast variables with the compact improved closure

Using the improved closure (3.14) in line with Proposition 2, which we refer to as the reduced improved closure, we find that

A.1 $$\begin{aligned}{}[ASI]&=(n-1)\left( (1-\phi )\frac{[AS][SI]}{n[S]}+\phi \frac{[AS][SI][IA]}{[A]\sum _{a}[aS][aI]/[a]}\right) \end{aligned}$$

A.2 $$\begin{aligned}&=(n-1)\left( (1-\phi )\frac{[AS][SI]}{n[S]}+\phi \frac{[AS][SI][IA]}{[A]\left( \frac{[SS][SI]}{[S]}+\frac{[SI][II]}{[I]}\right) }\right) . \end{aligned}$$

Using Eq. (A.2) to close the original pairwise Eqs. (2.2)–(2.6), we obtain the following system of equations:

A.3 $$\begin{aligned} \dot{[S]}= & {} -\tau [SI] \end{aligned}$$

A.4 $$\begin{aligned} \dot{[I]}= & {} \tau [SI]-\gamma [I] \end{aligned}$$

A.5 $$\begin{aligned} \dot{[SI]}= & {} -(\tau +\gamma )[SI]+\tau (n-1)\left( (1-\phi ) \frac{[SS][SI]}{n[S]}+\phi \frac{[I][SS][SI]}{[I][SS]+[S][II]}\right) \nonumber \\&-\,\tau (n-1)\left( (1-\phi )\frac{[SI]^{2}}{n[S]}+\phi \frac{[S][SI][II]}{[I][SS]+[S][II]}\right) \end{aligned}$$

A.6 $$\begin{aligned} \dot{[SS]}= & {} -2\tau (n-1)\left( (1-\phi )\frac{[SS][SI]}{n[S]}+ \phi \frac{[I][SS][SI]}{[I][SS]+[S][II]}\right) \end{aligned}$$

A.7 $$\begin{aligned} \dot{[II]}= & {} 2\tau [SI]-2\gamma [II] \nonumber \\&+\,2\tau (n-1)\left( (1-\phi ) \frac{[SI]^{2}}{n[S]}+\phi \frac{[S][SI][II]}{[I][SS]+[S][II]}\right) . \end{aligned}$$

As we have shown in the main body of the paper, the computation of the threshold requires a system of differential equations for the fast variables $\alpha =[SI]/[I]$ and $\delta =[II]/[I]$. We find

$$\begin{aligned} \frac{d\alpha }{dt}=\frac{[SI]^{\prime }}{[I]}-\frac{[SI][I]^{\prime }}{[I]^{2}} \end{aligned}$$

and substituting $[SI]^{\prime }$ from Eq. (A.5) and $[I]^{\prime }$ from Eq. (A.4), we obtain

A.8 $$\begin{aligned} \frac{d\alpha }{dt}&=-(\tau +\gamma )\frac{[SI]}{[I]}+\tau (n-1) \left( (1-\phi )\frac{[SS][SI]}{n[S][I]}+\phi \frac{[SS][SI]}{[I][SS]+[S][II]}\right) \nonumber \\&\quad -\tau (n-1)\left( (1-\phi )\frac{[SI]^{2}}{n[S][I]}+\phi \frac{[S][SI][II]}{[I]^{2}[SS]+[S][I][II]}\right) -\tau \frac{[SI]^{2}}{[I]^{2}}+\gamma \frac{[SI]}{[I]}. \end{aligned}$$

Replacing all $\frac{[SI]}{[I]}$ terms by $\alpha$ and all $\frac{[II]}{[I]}$ terms by $\delta$ gives

A.9 $$\begin{aligned} \frac{d\alpha }{dt}&=-(\tau +\gamma )\alpha +\tau (n-1)\left( (1-\phi ) \frac{[SS]}{n[S]}\alpha +\phi \alpha \frac{[SS]}{[SS]+[S]\delta }\right) \nonumber \\&\quad -\tau (n-1)\left( (1-\phi )\frac{[SI]}{n[S]}\alpha +\phi \alpha \delta \frac{[S]}{[SS]+[S]\delta }\right) -\tau \alpha ^{2}+\gamma \alpha , \end{aligned}$$

and evaluating $\frac{d\alpha }{dt}$ at the disease-free steady state $([S],[I],[SI],[SS],[II])=(N,0,0,nN,0)$ (B.1) gives

A.10 $$\begin{aligned} \frac{d\alpha }{dt}&=-(\tau +\gamma )\alpha +\tau (n-1)\left( (1-\phi ) \alpha +\phi \alpha \frac{nN}{nN+N\delta }\right) \nonumber \\&\quad -\tau (n-1)\left( \phi \alpha \delta \frac{N}{nN+N\delta }\right) -\tau \alpha ^{2}+\gamma \alpha . \end{aligned}$$

After simplification we find that

A.11 $$\begin{aligned} \frac{d\alpha }{dt}= & {} -\tau \alpha +\tau (n-1)\left( (1-\phi )\alpha + \phi \alpha \frac{n}{n+\delta }-\phi \alpha \delta \frac{1}{n+ \delta }\right) -\tau \alpha ^{2} \end{aligned}$$

A.12 $$\begin{aligned}= & {} -\tau \alpha +\tau (n-1)\left( (1-\phi )\alpha +\phi \alpha \left( \frac{n-\delta }{n+\delta }\right) \right) -\tau \alpha ^{2}. \end{aligned}$$

Differentiating $$\delta =\frac{[II]}{[I]}$$ gives

$$\begin{aligned} \frac{d\delta }{dt}=\frac{[II]^{\prime }}{[I]}-\frac{[II][I]^{\prime }}{[I]^{2}}, \end{aligned}$$

and substituting $[II]^{\prime }$ from Eq. (A.7) and $[I]^{\prime }$ from Eq. (A.4), we obtain

A.13 $$\begin{aligned} \frac{d\delta }{dt}&=2\tau \frac{[SI]}{[I]}-2\gamma \frac{[II]}{[I]}+ 2\tau (n-1)\left( (1-\phi )\frac{[SI]^{2}}{n[S][I]}+\phi \frac{[S][SI] [II]}{[I]^{2}[SS]+[S][I][II]}\right) \nonumber \\&\quad -\tau \frac{[SI][II]}{[I]^{2}}+ \gamma \frac{[II]}{[I]}. \end{aligned}$$

Replacing all $\frac{[SI]}{[I]}$ terms by $\alpha$ and all $\frac{[II]}{[I]}$ terms by $\delta$ gives

$$\begin{aligned} \frac{d\delta }{dt}= & {} 2\tau \alpha -2\gamma \delta +2\tau (n-1) \left( (1-\phi )\frac{[SI]}{n[S]}\alpha +\phi \alpha \delta \frac{[S]}{[SS]+[S]\delta }\right) -\tau \alpha \delta +\gamma \delta \\= & {} 2\tau \alpha -\gamma \delta +2\tau (n-1)\left( (1-\phi ) \frac{[SI]}{n[S]}\alpha +\phi \alpha \delta \frac{[S]}{[SS]+ [S]\delta }\right) -\tau \alpha \delta , \end{aligned}$$

and evaluating $\frac{d\delta }{dt}$ at the disease-free steady state (B.1) gives

A.14 $$\begin{aligned} \frac{d\delta }{dt}= & {} 2\tau \alpha -\gamma \delta +2\tau (n-1) \left( \phi \alpha \delta \frac{N}{nN+N\delta }\right) -\tau \alpha \delta \end{aligned}$$

A.15 $$\begin{aligned}= & {} 2\tau \alpha -\gamma \delta +2\tau (n-1)\left( \phi \alpha \delta \frac{1}{n+\delta }\right) -\tau \alpha \delta . \end{aligned}$$

Combining the differential equations for both $\alpha =\frac{[SI]}{[I]}$ and $\delta =\frac{[II]}{[I]}$, we have

A.16 $$\begin{aligned} \frac{d\alpha }{dt}= & {} -\tau \alpha +\tau (n-1)\left( (1-\phi )\alpha + \phi \alpha \left( \frac{n-\delta }{n+\delta }\right) \right) -\tau \alpha ^{2} \end{aligned}$$

A.17 $$\begin{aligned} \frac{d\delta }{dt}= & {} 2\tau \alpha -\gamma \delta +2\tau (n-1) \left( \frac{\phi \alpha \delta }{n+\delta }\right) -\tau \alpha \delta . \end{aligned}$$

## Standard linear-stability analysis for the case of the simple closure

An alternative way to determine the epidemic threshold is to consider the stability of the disease-free steady state

B.1 $$\begin{aligned} ([S],[I],[SI],[SS],[II])=(N,0,0,nN,0). \end{aligned}$$

When the disease-free steady state is stable, the system will always end up at the disease-free steady state and thus no epidemic will occur. When the disease-free steady state becomes unstable, there exists (at least) a second steady state whereby an epidemic will occur and [*S*] will no longer be equal to *N*. To determine a stability condition for the disease-free steady state (B.1), we must compute the Jacobian matrix *J* of the system (4.1)–(4.5), evaluated at the disease-free steady state, and solve to find its eigenvalues.

By computing partial derivatives of each differential equation (4.1)–(4.5) with respect to each model variable [*S*], [*I*], [*SI*], [*SS*] and [*II*], and evaluating each expression at the disease-free steady state (B.1), we obtain

B.2 $$\begin{aligned} J_{df}=\begin{pmatrix} 0 &{} \qquad 0 &{}\qquad -\tau &{} \qquad 0 &{}\qquad 0 \\ 0 &{}\qquad -\gamma &{}\qquad \tau &{}\qquad 0 &{}\qquad 0 \\ 0 &{}\qquad \frac{\partial \dot{[SI]}}{\partial [I]} &{}\qquad \frac{\partial \dot{[SI]}}{\partial [SI]} &{}\qquad 0 &{}\qquad \frac{\partial \dot{[SI]}}{\partial [II]} \\ 0 &{}\qquad \frac{\partial \dot{[SS]}}{\partial [I]} &{}\qquad \frac{\partial \dot{[SS]}}{\partial [SI]} &{}\qquad 0 &{}\qquad 0 \\ 0 &{}\qquad \frac{\partial \dot{[II]}}{\partial [I]} &{}\qquad \frac{\partial \dot{[II]}}{\partial [SI]} &{}\qquad 0 &{}\qquad \frac{\partial \dot{[II]}}{\partial [II]} \end{pmatrix}, \end{aligned}$$

with $\frac{\partial \dot{[SI]}}{\partial [I]}= \tau \xi \phi \left( \frac{2[SI]^{2}[II]}{n[I]^{3}}-\frac{[SI]^{2}}{[I]^{2}}\right)$, $\frac{\partial \dot{[SI]}}{\partial [SI]}= -(\tau +\gamma )+\tau \xi (1-\phi )n+ 2\tau \xi \phi \left( \frac{[SI]}{[I]}-\frac{[SI][II]}{n[I]^{2}}\right)$, $\frac{\partial \dot{[SI]}}{\partial [II]}=-\tau \xi \phi \frac{[SI]^{2}}{n[I]^{2}}$, $\frac{\partial \dot{[SS]}}{\partial [I]}=2\tau \xi \phi \frac{[SI]^{2}}{[I]^{2}}$, $\frac{\partial \dot{[SS]}}{\partial [SI]}=-2\tau \xi (1-\phi )n-4\tau \xi \phi \frac{[SI]}{[I]}$, $\frac{\partial \dot{[II]}}{\partial [I]}=-4\tau \xi \phi \frac{[SI]^{2}[II]}{n[I]^{3}}$, $\frac{\partial \dot{[II]}}{\partial [SI]}=2\tau +4\tau \xi \phi \frac{[SI][II]}{n[I]^{2}}$ and $\frac{\partial \dot{[II]}}{\partial [II]}= -2\gamma +2\tau \xi \phi \frac{[SI]^{2}}{n[I]^{2}}$ all containing variables $\frac{[SI]}{[I]}$ and $\frac{[II]}{[I]}$. The zero entries in $J_{df}$ reflect the true values that the respective partial derivatives attain at the disease-free equilibrium. However, the majority of the non-zero matrix entries involve $\frac{[SI]}{[I]}$ and $\frac{[II]}{[I]}$. Since $[I]=[SI]=[II]=0$ at the disease-free steady state, both of these quantities are ill-defined. Hence, not all entries of the Jacobian can be evaluated at the equilibrium. This issue prevents the computation of the eigenvalues of $J_{df}$ and thus the value of the epidemic threshold. In order to progress, we need to determine the correct values for $\alpha =\frac{[SI]}{[I]}$ and $\delta =\frac{[II]}{[I]}$. We note that the correct value of $\alpha =\frac{[SI]}{[I]}$ is also required in Eq. (4.10), and the threshold cannot be computed without it.

In fact, using only $\phi =0$, the Jacobian at the disease-free steady state (B.1) becomes

B.3 $$\begin{aligned} J_{df\_no\_clust}=\begin{pmatrix} 0 &{}\qquad 0 &{}\qquad -\tau &{}\qquad 0 &{}\qquad 0 \\ 0 &{}\qquad -\gamma &{}\qquad \tau &{} \qquad 0 &{} \qquad 0 \\ 0 &{}\qquad 0 &{}\qquad -\gamma +\tau (n-2) &{} \qquad 0 &{} \qquad 0 \\ 0 &{}\qquad 0 &{}\qquad -2\tau (n-1) &{}\qquad 0 &{}\qquad 0 \\ 0 &{}\qquad 0 &{}\qquad 2\tau &{}\qquad 0 &{}\qquad -2\gamma \end{pmatrix}. \end{aligned}$$

It is straightforward to show that the eigenvalues are given by $\lambda _{1}=0$, $\lambda _{2}=-\gamma$, $\lambda _{3}=\tau (n-2)-\gamma$, $\lambda _{4}=0$ and $\lambda _{5}=-2\gamma$. The only eigenvalue that can be non-zero and non-negative is $\lambda _{3}=\tau (n-2)-\gamma$. Hence, we know that the disease-free steady state (B.1) is stable when $\lambda _{3}\le 0$ and becomes unstable when $\lambda _{3}>0$. Thus, the epidemic threshold is given by $\lambda _{3}=0$ and this can be rearranged to give $\tau (n-2)/\gamma =1$. This is equivalent to the calculation based on determining the quasi-equilibrium of the fast variables.

## Standard linear-stability analysis for the case of the compact improved closure

To determine an epidemic threshold, we consider conditions for stability of the disease-free steady state (B.1). To do so, we compute the Jacobian matrix evaluated at the disease-free steady state as

C.1 $$\begin{aligned} J_{df_2}=\begin{pmatrix} 0 &{} \qquad 0 &{} \qquad -\tau &{}\qquad 0 &{} \qquad 0 \\ 0 &{}\qquad -\gamma &{}\qquad \tau &{}\qquad 0 &{}\qquad 0\\ 0 &{} \qquad \frac{\partial \dot{[SI]}}{\partial [I]} &{}\qquad \frac{\partial \dot{[SI]}}{\partial [SI]} &{}\qquad 0 &{}\qquad \frac{\partial \dot{[SI]}}{\partial [II]} \\ 0 &{} \qquad \frac{\partial \dot{[SS]}}{\partial [I]} &{}\qquad \frac{\partial \dot{[SS]}}{\partial [SI]} &{}\qquad 0 &{}\qquad \frac{\partial \dot{[SS]}}{\partial [II]} \\ 0 &{}\qquad \frac{\partial \dot{[II]}}{\partial [I]} &{}\qquad \frac{\partial \dot{[II]}}{\partial [SI]} &{}\qquad 0 &{}\qquad \frac{\partial \dot{[II]}}{\partial [II]} \end{pmatrix} \end{aligned}$$

where $\frac{\partial \dot{[SI]}}{\partial [I]}= 2\tau (n-1)\phi \alpha \delta \frac{n}{n^{2}+2n\delta +\delta ^{2}}$, $\frac{\partial \dot{[SI]}}{\partial [SI]}= -(\tau +\gamma )+\tau (n-1)\Big ((1-\phi )+\phi \left( \frac{n-\delta }{n+\delta }\right) \Big )$, $\frac{\partial \dot{[SI]}}{\partial [II]}= -2\tau (n-1)\left( \phi \alpha \frac{n}{n^{2}+2n\delta +\delta ^{2}}\right)$, $\frac{\partial \dot{[SS]}}{\partial [I]}= -2\tau (n-1)\left( \phi \alpha \delta \frac{n}{n^{2}+2n\delta +\delta ^{2}}\right)$, $\frac{\partial \dot{[SS]}}{\partial [SI]}= -2\tau (n-1)\left( (1-\phi )+\phi \frac{n}{n+\delta }\right)$, $\frac{\partial \dot{[SS]}}{\partial [II]}= 2\tau (n-1)\left( \phi \alpha \frac{n}{n^{2}+2n\delta +\delta ^{2}}\right)$, $\frac{\partial \dot{[II]}}{\partial [I]}= -2\tau (n-1)\left( \phi \alpha \delta \frac{n}{n^{2}+2n\delta +\delta ^{2}}\right)$, $\frac{\partial \dot{[II]}}{\partial [SI]}= 2\tau +2\tau (n-1)\left( \phi \delta \frac{1}{n+\delta }\right)$ and $\frac{\partial \dot{[II]}}{\partial [II]}=-2\gamma +2\tau (n-1)\left( \phi \alpha \frac{n}{n^{2}+2n\delta +\delta ^{2}}\right)$ cannot be fully evaluated as they contain products of the problematic variables $\alpha =\frac{[SI]}{[I]}$ and $\delta =\frac{[II]}{[I]}$.

The Jacobian above becomes useful once analytical expressions for $\alpha$ and $\delta$ are obtained (or it could be an asymptotic expansion or even numerical values). Plugging these in the Jacobian will allow to either numerically or analytically compute the threshold. We note that using the linear-stability analysis or focusing on the initial growth rate should lead to the same threshold value, as was already shown for the case of the system with the simple closure in Sect. B.
